# Interactions between the Hepatitis C Virus Nonstructural 2 Protein and Host Adaptor Proteins 1 and 4 Orchestrate Virus Release

**DOI:** 10.1128/mBio.02233-17

**Published:** 2018-03-13

**Authors:** Fei Xiao, Stanley Wang, Rina Barouch-Bentov, Gregory Neveu, Szuyuan Pu, Melanie Beer, Stanford Schor, Sathish Kumar, Vlad Nicolaescu, Brett D. Lindenbach, Glenn Randall, Shirit Einav

**Affiliations:** a Division of Infectious Diseases and Geographic Medicine, Department of Medicine, and Department of Microbiology and Immunology, Stanford University School of Medicine, Stanford, California, USA; b Department of Microbiology, the University of Chicago, Chicago, Illinois, USA; c Department of Microbial Pathogenesis, Yale School of Medicine, New Haven, Connecticut, USA; Twincore; Johns Hopkins Bloomberg School of Public Health

**Keywords:** adaptor proteins, antiviral strategies, cell-to-cell spread, hepatitis C virus, intracellular membrane trafficking, viral release, virus-host interactions

## Abstract

Hepatitis C virus (HCV) spreads via secreted cell-free particles or direct cell-to-cell transmission. Yet, virus-host determinants governing differential intracellular trafficking of cell-free- and cell-to-cell-transmitted virus remain unknown. The host adaptor proteins (APs) AP-1A, AP-1B, and AP-4 traffic in post-Golgi compartments, and the latter two are implicated in basolateral sorting. We reported that AP-1A mediates HCV trafficking during release, whereas the endocytic adaptor AP-2 mediates entry and assembly. We demonstrated that the host kinases AAK1 and GAK regulate HCV infection by controlling these clathrin-associated APs. Here, we sought to define the roles of AP-4, a clathrin-independent adaptor; AP-1A; and AP-1B in HCV infection. We screened for interactions between HCV proteins and the μ subunits of AP-1A, AP-1B, and AP-4 by mammalian cell-based protein fragment complementation assays. The nonstructural 2 (NS2) protein emerged as an interactor of these adaptors in this screening and by coimmunoprecipitations in HCV-infected cells. Two previously unrecognized dileucine-based motifs in the NS2 C terminus mediated AP binding and HCV release. Infectivity and coculture assays demonstrated that while all three adaptors mediate HCV release and cell-free spread, AP-1B and AP-4, but not AP-1A, mediate cell-to-cell spread. Live-cell imaging revealed HCV cotrafficking with AP-1A, AP-1B, and AP-4 and that AP-4 mediates HCV trafficking in a post-Golgi compartment. Lastly, HCV cell-to-cell spread was regulated by AAK1 and GAK and thus susceptible to treatment with AAK1 and GAK inhibitors. These data provide a mechanistic understanding of HCV trafficking in distinct release pathways and reveal a requirement for APs in cell-to-cell viral spread.

## IMPORTANCE

HCV spreads via cell-free infection or cell-to-cell contact that shields it from antibody neutralization, thereby facilitating viral persistence. Yet, factors governing this differential sorting remain unknown. By integrating proteomic, RNA interference, genetic, live-cell imaging, and pharmacological approaches, we uncover differential coopting of host adaptor proteins (APs) to mediate HCV traffic at distinct late steps of the viral life cycle. We reported that AP-1A and AP-2 mediate HCV trafficking during release and assembly, respectively. Here, we demonstrate that dileucine motifs in the NS2 protein mediate AP-1A, AP-1B, and AP-4 binding and cell-free virus release. Moreover, we reveal that AP-4, an adaptor not previously implicated in viral infections, mediates cell-to-cell spread and HCV trafficking. Lastly, we demonstrate cell-to-cell spread regulation by AAK1 and GAK, host kinases controlling APs, and susceptibility to their inhibitors. This study provides mechanistic insights into virus-host determinants that facilitate HCV trafficking, with potential implications for pathogenesis and antiviral agent design.

## INTRODUCTION

Hepatitis C virus (HCV) is a major global health problem; it is estimated to chronically infect 71 million people worldwide (1). HCV persists in the majority (>70%) of infected individuals, resulting in severe liver disease, including cirrhosis, liver failure, and hepatocellular carcinoma (reviewed in reference 2).

HCV is an enveloped, positive single-stranded RNA virus in the *Flaviviridae* family. Its 9.6-kb genome encodes a single polyprotein that is proteolytically cleaved into individual proteins. The HCV core protein and E1 and E2 glycoproteins form new virions; nonstructural (NS) proteins NS3, -4A, -4B, -5A, and -5B form the viral replication machinery, whereas p7 and NS2 are essential for infectious virus production (3–5). The current model of infectious HCV production suggests that viral particles begin to assemble on or near the surface of lipid droplets (LD), where core is concentrated (6). Similar to flaviviruses, HCV is thought to bud into the endoplasmic reticulum (ER), where the envelope glycoproteins are retained. HCV particles, rendered infectious upon budding, exit the cell via the secretory pathway (7), where they cotraffic with various components of the ER, the trans-Golgi network (TGN), and recycling endosomes (8). Upon their release, these cell-free viral particles can infect distant cells. Infectious HCV production requires coordination of all 10 HCV proteins along with multiple host factors (6). NS2, in particular, plays a critical role in early viral assembly, envelopment, maturation, and release (3, 4, 9–11). Nevertheless, a comprehensive understanding of the mechanisms that govern viral particle trafficking during HCV release is still lacking.

In addition to the release of cell-free virus, HCV transmission occurs via cell-to-cell spread, whereby viral particles spread directly to neighboring cells while being protected from antibody neutralization and other extracellular viral clearance mechanisms (12–14). Cell-to-cell spread is therefore implicated in immune evasion, HCV persistence, and antiviral treatment failure (15, 16). It remains unknown, however, how viral particles are differentially directed to cell membrane sites for cell-to-cell versus cell-free spread.

Intracellular membrane traffic relies, to a large extent, on the interactions between adaptor protein (AP) complexes (AP-1 through AP-5) and the transmembrane cargo (17). APs are heterotetrameric complexes composed of two large (β and α, γ, δ, or ε) subunits (110 to 130 kDa), a medium (μ) subunit (~50 kDa), and a small (σ) subunit (15 to 20 kDa) (17). AP complexes orchestrate the formation of vesicles destined for transport by distinct intracellular pathways. While AP-2 sorts in the endocytic pathway, AP-1 and AP-4 facilitate sorting in post-Golgi compartments (18, 19). Specifically, AP-1A typically mediates sorting from the TGN to recycling endosomes; AP-1B mediates sorting from the TGN to the basolateral membrane, whereas AP-4 is thought to facilitate exiting from the TGN and sorting by both the endosomal and basolateral pathways (17, 20–24). Recognition of either tyrosine-based (YXXØ) or dileucine-based [(D/E)XXXL(L/I) and (LL/LI)] motifs within the cargo protein by subunits of the AP complex mediates these interactions (X is any amino acid, and Ø is a bulky hydrophobic amino acid) (19). The two host cell kinases AP-2-associated protein kinase 1 (AAK1) and cyclin G-associated kinase (GAK) regulate receptor-mediated endocytosis and TGN transport (25, 26). Specifically, AAK1 and GAK phosphorylate the μ subunits of clathrin-associated AP-1 and AP-2, thereby enhancing their binding to sorting motifs within the cargo (25, 27–29). Moreover, GAK recruits clathrin-associated APs to the plasma membrane and TGN (30).

The clathrin-associated AP-1 and AP-2 complexes have been implicated in multiple viral infections (31–34). We have previously reported that AP-2 is essential for HCV entry (35) and is also recruited by a tyrosine-based motif within the HCV core protein to LDs, where it plays a critical role in HCV assembly (36). More recently, we and others have demonstrated a role for AP-1A in HCV release (8, 37–40). By using live-cell imaging, we showed that a fraction of HCV particles cotraffics with AP-1A and AP-2 (39). Moreover, we showed that AAK1 and GAK regulate HCV entry and assembly via AP-2 phosphorylation and HCV release via AP-1A phosphorylation (35, 36, 39). Compounds with potent anti-AAK1 and/or anti-GAK activity, including the already approved anticancer drugs sunitinib and erlotinib and novel more selective AAK1 and GAK inhibitors, inhibit HCV entry, assembly, and cotrafficking with AP-1A and AP-2 (35, 36, 39, 41). Nevertheless, the HCV determinant(s) required for binding of APs for steps beyond viral assembly remained unknown. Moreover, the role of AP complexes in viral cell-to-cell spread and the role of AP-4 specifically in any aspect of a viral life cycle have not been reported to date.

We hypothesized that HCV proteins differentially bind AP complexes via tyrosine- or dileucine-based motifs to mediate intracellular traffic of viral particles at temporally distinct late steps of the viral life cycle. To test this hypothesis, we screened for interactions between HCV proteins and the μ subunits of four AP complexes by mammalian cell-based protein fragment complementation assays (PCAs) (36, 42). NS2 emerged as a critical viral protein for binding of the AP-1A, AP-1B, and AP-4 complexes. We demonstrate that two heretofore unrecognized dileucine-based motifs in the C-terminal protease domain of NS2 mediate binding to APs and HCV release. Furthermore, we show that while AP-1A, AP-1B, and AP-4 are required for HCV release and cell-free infectivity, AP-1B and AP-4, but not AP-1A, are involved in the mediation of cell-to-cell spread. Importantly, we provide evidence that the majority of HCV particles cotraffic with AP-4, in part via a post-Golgi pathway. Lastly, we demonstrate a role for AAK1- and GAK-regulated AP activity in yet another step of the HCV life cycle beyond entry, assembly, and cell-free virus release, namely, cell-to-cell spread.

## RESULTS

### Differential binding of AP complexes by HCV proteins.

**FIG 1:**
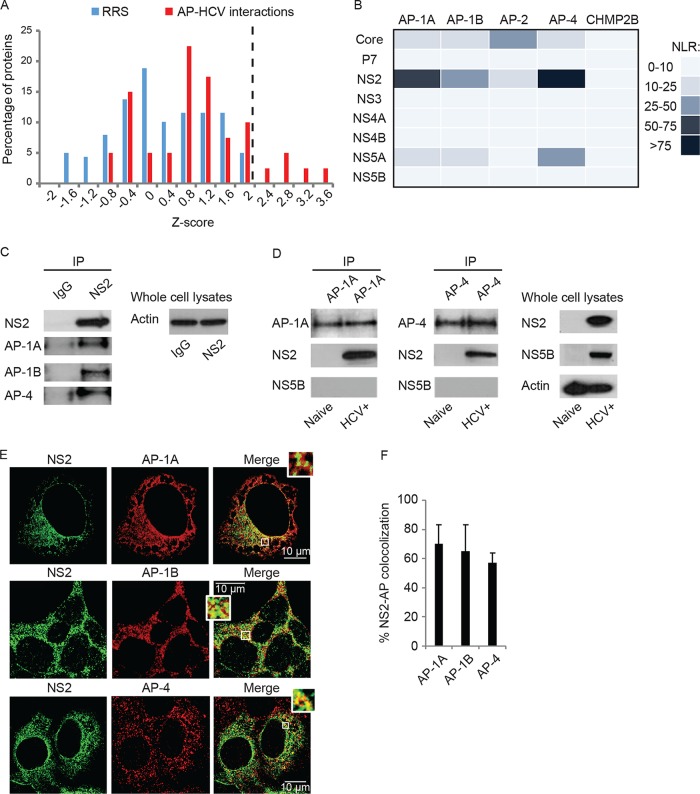
Interactions between HCV proteins and host APs. (A) Results of PCA screening for interactions of AP μ subunits with individual HCV proteins. Histogram of the mean *z* scores of the set studied and RRS of interactions obtained from three independent experiments. The dotted line defines the cutoff used for positive interactions. (B) Heat map of the interactions color coded on the basis of the NLR. (C) NS2 interacts with APs in HCV RNA-transfected cells. Immunoprecipitations (IPs) from membrane fractions of HCV RNA-transfected or naive Huh7.5 cells with anti-NS2 (C), anti-AP-1A, or anti-AP-4 (D) antibodies and IgG controls (C). Antibodies used for immunoblotting are indicated on the left. (E) Representative confocal IF microscopy images at ×40 magnification of AP (red) and NS2 (green) in HCV-transfected cells. Scale bars represent 10 µm. (F) Quantitative colocalization analysis of *z* stacks by using Manders’ colocalization coefficients. Mean M2 values are presented as percent colocalization (the fraction of green intensity that coincides with red intensity) ± SD.

We initially screened for interactions between the AP-1A, AP-1B, AP-2, and AP-4 complexes and the HCV proteome (excluding E1 and E2, which form disulfide-linked misfolded aggregates when ectopically coexpressed in cells [43]) by using PCAs. This PCA format relies on reversible reconstitution of a split luciferase reporter and provides a high-fidelity means to measure weak and transient interactions (36) such as those between APs and cargo (*K*_*d*_s in the micromolar range) (18, 24). Moreover, it allows detection of interactions involving membrane proteins in mammalian cells and within appropriate subcellular compartments (36, 42). Since the μ subunits of AP complexes interact with both tyrosine and dileucine motifs (31–34), their coding genes were fused to an N-terminal luciferase fragment reporter (GLuc1-A), while individual HCV proteins derived from the J6/JFH genome (44) were fused to an N-terminal complementary luciferase fragment (GLuc2-B). AP and viral genes were transfected pairwise into 293T cells. When screening for NS3-AP interactions, a plasmid encoding FLAG-tagged NS4A was added to allow membrane binding of the NS3 protein (45). Expression of the viral (42) and host APs (see Fig. S1A in the supplemental material) was confirmed by Western blot assays. Luciferase activity was measured at 24 h posttransfection, and results were expressed as normalized luminescence ratios (NLR). We benchmarked the accuracy and sensitivity of this screening by a random reference set (RRS) composed of 53 noninteracting human protein pairs and CHMP2B, which did not interact with the HCV proteome in a recent unrelated screening (42) (Fig. S1B). *z* scores indicating the number of standard deviations (SDs) above the mean NLR of the control RRS were calculated. A histogram distribution curve of the mean *z* score values obtained from three independent experiments exhibited a clear separation between the set studied and the RRS (*P* = 6.22 × 10^−6^, *t* test) (Fig. 1A). A cutoff value of >2.2 SDs (corresponding to an NLR of >25) was chosen as the threshold to define positive interactions. Novel prominent interactions between NS2 and several APs were identified in this screening. NS2 bound the μ subunit of AP-4 with the greatest apparent affinity, followed by AP-1A and AP-1B (Fig. 1B). NS5A also bound AP-4, albeit with a lower apparent affinity than NS2. In contrast, HCV core bound AP-2 with the highest apparent affinity, supporting the important role of this interaction in HCV infection (36) (Fig. 1B). P7, NS3, NS4A, NS4B, and NS5B coexpression with APs yielded luciferase signals comparable to the background (Fig. 1B). Moreover, NS2 did not bind the μ subunits of the AP-2, AP-3, and AP-5 complexes, supporting the specificity of its interactions with the AP-1A, AP-1B, and AP-4 complexes (Fig. S1C).

10.1128/mBio.02233-17.2 FIG S1  Differential binding of AP complexes by HCV proteins. (A) Levels of APs in lysates of cells transfected with the plasmids indicated and blotted with anti-GLuc and anti-actin antibodies. (B) Examples of protein interactions included in the RRS tested via PCA. The first four columns list the genes and accession numbers of the interactors (A and B). The fifth column lists the average *z* scores measured by PCA in three independent experiments, each in triplicate. (C) NS2 binding to the μ subunits of the AP complexes indicated via PCA. Mean values and SDs of three independent experiments are shown. The dotted line represents the cutoff for positivity (NLR, >25) derived from the histogram shown in Fig. 1. (D) IPs from membrane fractions of Huh7.5 cells ectopically expressing WT or quadruple dileucine GLuc-NS2 mutant (QM) and AP-4 with anti-AP-4 antibodies and IgG controls. Membranes were blotted with antibodies against AP-4, GLuc (NS2), and actin. Download FIG S1, TIF file, 0.7 MB. Copyright © 2018 Xiao et al. 2018 Xiao et al. This content is distributed under the terms of the Creative Commons Attribution 4.0 International license.

### NS2 binds AP-1 and AP-4 in the context of HCV infection.

Since NS2 emerged as a key binding partner in the screening, we validated its interactions with AP-1A, AP-1B, and AP-4. Coimmunoprecipitations (co-IPs) were conducted with membrane fractions derived from cells transfected with J6/JFH HCV RNA and untransfected (naive) controls. Because of the weak and transient nature of typical adaptor-cargo interactions (18, 24), a cross-linker was added to allow covalent binding of the already bound interacting proteins, as we previously reported (36, 42). Anti-NS2 antibody effectively pulled down AP-1A, AP-1B, and AP-4, whereas only a background signal was demonstrated with IgG controls (Fig. 1C). Reciprocal co-IPs revealed that anti-AP-1A or -AP-4 antibodies pulled down NS2 but not a control protein, NS5B, from HCV RNA-transfected cells (Fig. 1D). Lack of NS2 signal in IPs from naive cells (Fig. 1D) confirmed the specificity of viral protein detection in the HCV RNA-transfected cells. In addition, significant colocalization of NS2 with AP-1A, AP-1B, and AP-4 was observed by confocal immunofluorescence (IF) analysis of 15 to 20 HCV-infected cells (Manders’ colocalization coefficients of 70% ± 13%, 65% ± 18%, and 57% ± 7%, respectively) (Fig. 1E and F).

### NS2 harbors dileucine motifs that mediate AP-1 and AP-4 binding and HCV release.

**FIG 2:**
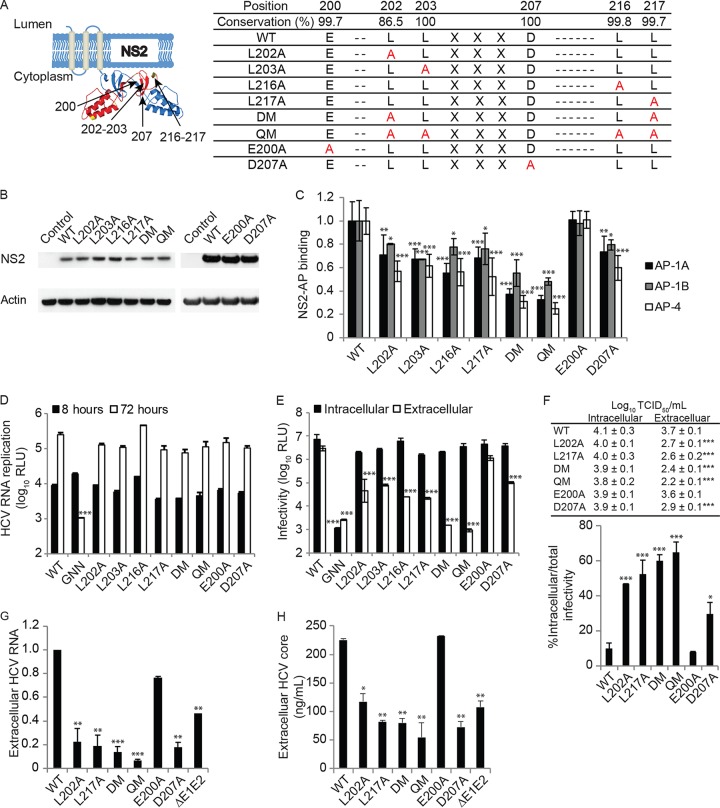
NS2 harbors two dileucine motifs that mediate AP binding and HCV release. (A) Dileucine motifs in the C terminus of NS2 and the cloned mutations. The schematic shown was based on data from reference 46. (B) Levels of NS2 in lysates of cells transfected with the plasmids indicated and blotted with anti-GLuc and anti-actin antibodies. (C) Interactions of WT or mutant NS2 with AP-1A, AP-1B, and AP-4 by PCAs. Plotted are NLRs relative to WT NS2-AP binding. (D) Cells were electroporated with WT or mutated NS2 bicistronic J6/H77NS2/JFH HCV RNA. HCV RNA replication measured via luciferase assays 8 and 72 h after HCV RNA electroporation. RLU, relative light units. (E) HCV infectivity measured via luciferase assays by inoculating naive cells with lysates (intracellular) and supernatants (extracellular) from electroporated cells. (F) Intra- and extracellular infectivity titers measured by limiting-dilution assays (top) and percentages of intracellular infectivity per total (intra- and extracellular) infectivity for the dish (bottom). Viral RNA (G) and HCV core protein (H) release into culture supernatant at 72 h postelectroporation measured by qRT-PCR and ELISA, respectively. GNN is a replication-incompetent HCV strain. ΔE1-E2 is an assembly-defective mutant. Results in panels C to H represent data pooled from at least two independent experiments each with 3 to 10 biological replicates. Shown are the mean ± SD. *, *P* < 0.05; **, *P* < 0.01; ***, *P* < 0.001 (relative to the corresponding WT; one-way [C and E to H] or two-way [D] ANOVA with Dunnett’s *post hoc* test).

Inspection of the primary sequence of NS2 revealed two conserved dileucine motifs (LL) within the cytoplasmic C-terminal protease domain of the protein (Fig. 2A) (10, 46). To study the role of these dileucine motifs in the binding of AP-1A, AP-1B, and AP-4, we first introduced single, double (double mutant [DM]; L202A-L217A), and quadruple (quadruple mutant [QM]; L202A-L203A-L216A-L217A) leucine-to-alanine substitutions into the GLuc-NS2 vector (Fig. 2A). None of these mutations impaired NS2 expression (Fig. 2B), as previously shown with respect to L217 mutations (9). The single mutations reduced AP-4 binding measured via PCAs by ~2-fold relative to wild-type (WT) NS2, whereas the DM and QM reduced AP-4 binding by 3- and 4-fold, respectively (Fig. 2C). These NS2 mutations also reduced AP-1A and AP-1B binding, albeit the magnitude of the effect was smaller (Fig. 2C). Additionally, QM NS2 reduced AP-4 binding relative to WT NS2 via co-IPs from membrane fractions of Huh7.5 cells ectopically expressing WT or QM mutant GLuc-NS2 and AP-4, supporting the PCA data (Fig. S1D).

Dileucine-based sorting motifs often harbor acidic residues in the −4 [(D/E)XXXL(L/I)], −3 (DXXLL) (19, 32–34), or other position (47–49). NS2 has two highly conserved acidic residues adjacent to the first leucine pair: glutamate in position −2 and aspartate in position +4 (Fig. 2A). To determine the roles of these acidic residues, we mutated them individually to alanine. E200A and D207A did not reduce NS2 expression (Fig. 2B). Whereas the E200A mutation had no effect on AP binding, the D207A mutation reduced NS2 binding to AP-1A, AP-1B, and AP-4 (Fig. 2C). No conserved acidic residues are present adjacent to the second leucine pair of NS2. These results suggest that AP-1A, AP-1B, and AP-4 binding is mediated by two dileucine motifs within the C terminus of NS2. Nevertheless, the remaining signal observed with the QM suggests that additional NS2 residues may be involved in the mediation of AP μ subunit binding.

To investigate the role of these NS2 residues in the HCV life cycle, we introduced the single, double, and quadruple leucine mutations and single acidic residue mutations into a bicistronic luciferase reporter HCV genome, J6/H77NS2/JFH(NS2-IRES-nsGluc2AUbi) (3), which enables expression of the HCV replicase independently of NS2-3 cleavage. These mutations did not impair HCV RNA replication, as measured by luciferase assays at 8 and 72 h posttransfection (Fig. 2D). Moreover, inoculation of naive cells with clarified cell lysates derived from the HCV RNA-transfected cells demonstrated no effect of the mutations on intracellular infectivity (Fig. 2E). Nevertheless, the single-leucine and D207A mutations, but not the E200A mutation, caused an up to ~2-log reduction in extracellular infectivity measured by inoculation of naive cells with supernatants derived from the HCV RNA-transfected cells, consistent with a defect in viral release (Fig. 2E). The extracellular infectivity of the DM and QM was at the background level (~3.5-log reduction). To determine whether this defect in viral release was associated with accumulation of intracellular particles, we measured infectious virus titers in cell lysates and culture supernatants derived from cells transfected with the WT or NS2 mutant HCV genome. Consistent with our luciferase data, NS2 mutations significantly reduced the extracellular, but not the intracellular, viral titer (Fig. 2F). Moreover, these mutations significantly increased the ratio of intracellular infectivity to total (intra- plus extracellular) infectivity (Fig. 2F). The magnitude of intracellular particle accumulation correlated with the effect of these mutations on viral release. We also measured the effect of these mutations on the release of noninfectious core protein- and RNA-containing particles. Detectable levels of HCV RNA and core protein release were measured in culture supernatants by quantitative reverse transcription (qRT)-PCR and enzyme-linked immunosorbent assay (ELISA), respectively (Fig. 2G and H), as previously described (11, 36). Nevertheless, the levels of defective particles released by the various NS2 mutants correlated with the infectious titers and were not higher than those released by the assembly-defective ΔE1-E2 mutant (Fig. 2G and H). Together, these results suggest that NS2 dileucine motifs mediate binding to AP-1A, AP-1B, and AP-4 and consequently HCV release.

### AP-1A, AP-1B, and AP-4 mediate HCV release.

**FIG 3:**
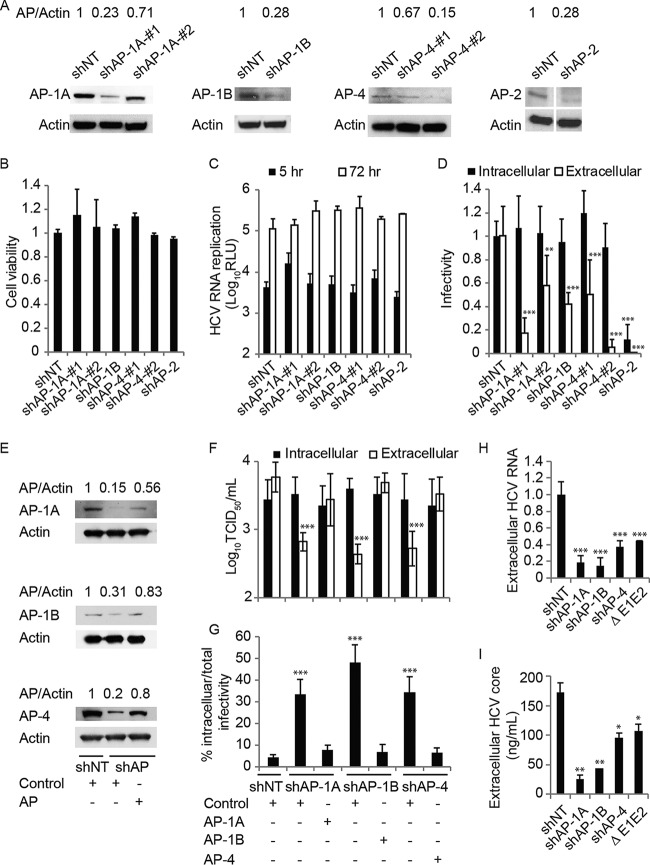
AP-1A/B and AP-4 mediate HCV release. (A) Confirmation of gene expression knockdown by Western blotting in Huh7.5 cells stably expressing AP shRNA or an NT control (values are AP-to-actin protein ratios relative to the NT control). (B) Relative cell viability in these cell lines measured by alamarBlue assays. (C) HCV RNA replication measured via luciferase assays 5 and 72 h after HCV RNA electroporation. RLU, relative light units. (D) HCV infectivity measured via luciferase assay by inoculating naive cells with lysates (intracellular) and supernatants (extracellular) from electroporated cells. (E to G) Levels of APs by Western blot analysis (E), intra- and extracellular viral titers measured by limiting-dilution assays (F), and intracellular infectivity as a percentage of the total infectivity (G) in cells concurrently transduced with shAP-1A, shAP-1B, and shAP-4 and transfected with the respective shRNA-resistant AP cDNA or an empty control plasmid. (H and I) Viral RNA (H) and core protein (I) release into the culture supernatant at 72 h postelectroporation measured by qRT-PCR and ELISA, respectively. Data are plotted relative to NT control values. Results in panels C, D, and F to I represent data pooled from three independent experiments each with three to six biological replicates. Shown are the mean ± SD. *, *P* < 0.05; **, *P* < 0.01; ***, *P* < 0.001 (relative to the corresponding NT control; one-way [B, D, and F to I] or two-way [C] ANOVA with Dunnett’s [C, D, H, and I] or Tukey’s [F and G] *post hoc* test).

We and others have previously demonstrated that the μ (AP1M1), σ (AP1S3), and γ (AP1G1) subunits of the AP-1A complex are required for HCV release (8, 37–40). Here, we sought to determine whether the AP-1B and AP-4 complexes, which emerged in the PCA screening as binding partners of NS2, are also required for infectious HCV production. To do so, we established Huh7.5 cell lines stably expressing short hairpin RNAs (shRNAs) targeting the various AP genes or a nontargeting (NT) sequence and transfected them with J6/JFH(p7-Rluc2A), a luciferase reporter virus, RNA (50). Effective suppression of APs was achieved (Fig. 3A) without apparent cytotoxic effects (Fig. 3B). AP-1A, AP-1B, AP-4, and AP-2 depletion had no effect on HCV RNA replication, as measured by luciferase assays at 5 and 72 h posttransfection (Fig. 3C). Inoculation of naive cells with clarified cell lysates derived from the HCV RNA-transfected (AP-depleted or NT control) cells resulted in comparable intracellular infectivity (Fig. 3D). Nevertheless, AP-1A, AP-1B, and AP-4 depletion caused a ~1-log reduction in extracellular infectivity upon inoculation of naive cells with supernatants derived from the HCV RNA-transfected cells. This effect on HCV release correlated with the level of AP-1A and AP-4 suppression. In contrast, as we previously reported (36), AP-2 depletion reduced both intra- and extracellular infectivity (Fig. 3D). Moreover, AP-1A, AP-1-B, and AP-4 depletion reduced the extracellular but not intracellular viral titer and significantly increased the accumulation of infectious intracellular viral particles (Fig. 3E to G). Importantly, ectopic expression of shRNA-resistant AP-1A, AP-1B, and AP-4 reversed the effect of the respective shRNAs on HCV release (Fig. 3E to G). Lastly, the observed defect in HCV release was not associated with increased release of noninfectious viral particles, as indicated by the levels of HCV RNA and core protein in supernatants derived from cells depleted of these APs (Fig. 3H and I). These results indicate that while AP-2 mediates HCV assembly, AP-1A, AP-1B, and AP-4 mediate HCV release.

### Differential requirement for host APs in distinct modes of HCV spread.

**FIG 4:**
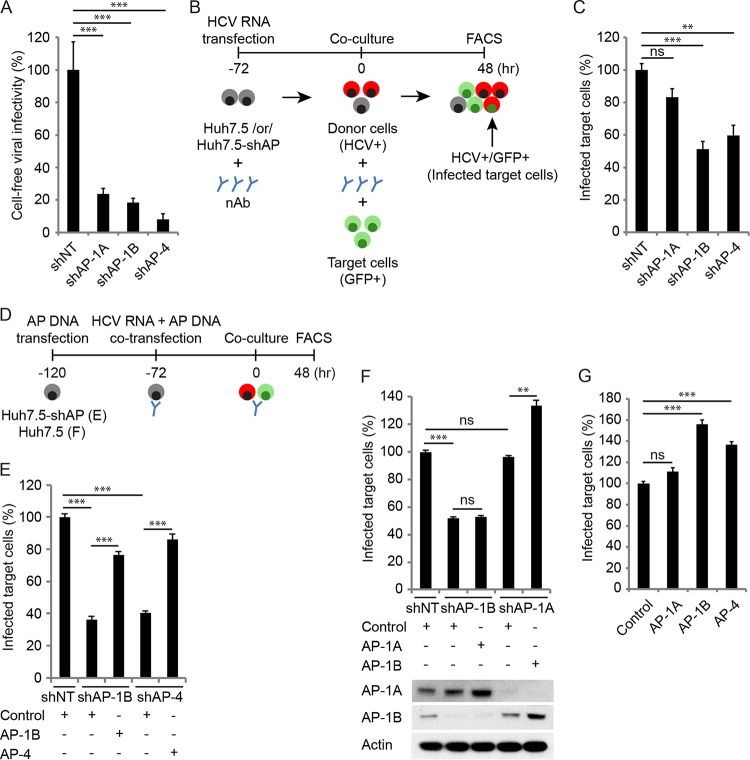
AP-1B and AP-4 mediate HCV cell-to-cell spread. (A) Cell-free infectivity measured via luciferase assays 6 h postinoculation of naive cells with supernatants derived from HCV RNA-transfected Huh7.5 cells stably expressing AP shRNA or an NT control. (B) Schematic of the coculture assay. (C) Cell-to-cell spread 48 h after coculturing of HCV RNA-transfected donor cells depleted of AP or NT controls with GFP-expressing target cells measured via FACS analysis following staining of HCV NS5A. Plotted is the percentage of infected target cells (NS5A^+^ GFP^+^) in the target cell population (GFP^+^) pooled from two independent experiments relative to the NT control. (D) Schematic of the experiments displayed in panels E to G. (E, F) Cell-to-cell spread (E and F, top) and expression (see Fig. 3E and F, bottom) in cells concurrently transduced with lentiviruses expressing the shRNAs indicated and transfected with shRNA-resistant GLuc-tagged AP-1A, AP-1B, or AP-4 or an empty control. (G) Cell-to-cell spread in Huh7.5 cells following transfection with the GLuc-tagged APs indicated or an empty control. ns, nonsignificant. Representative experiments of at least two conducted, each with three biological replicates, are shown. Shown are the mean ± SD. *, *P* < 0.05; **, *P* < 0.01; ***, *P* < 0.001 (relative to the corresponding NT control; one-way ANOVA with Dunnett’s [A, C, and G] or Tukey’s [E and F] *post hoc* test).

To determine whether AP-1A, AP-1B, and AP-4 specifically mediate the release of secreted and/or cell-to-cell-transmitted viruses, we first studied the effect of their depletion on cell-free infectivity. Stable cell lines depleted of AP-1A, AP-1B, or AP-4 and NT control cells (Fig. 3A) were electroporated with J6/JFH(p7-Rluc2A) RNA and incubated for 72 h. The infectivity of cell-free virus was measured in a single cycle, independently of cell-to-cell spread, via luciferase assays 6 h following the inoculation of naive Huh7.5 cells with culture supernatants derived from the electroporated cells. Depletion of AP-1A, AP-1B, and AP-4 significantly reduced cell-free infectivity relative to that of the NT control (Fig. 4A).

Next, we measured the effect of AP-1A, AP-1B, or AP-4 depletion on HCV cell-to-cell spread by coculture assays (14, 16, 51). Cell lines stably expressing shRNAs targeting AP-1A, AP-1B, AP-4, or an NT sequence (Fig. 3A) were electroporated with Jc1 HCV RNA (52). At 72 h posttransfection, these HCV donor cells were cocultured with green fluorescent protein (GFP)-expressing Huh7.5 target cells at a 1:2 ratio for 48 h. This was followed by immunostaining for HCV NS5A protein and quantification of the percentage of newly infected target cells (GFP^+^ NS5A^+^) in the target cell population (GFP^+^) by flow cytometry (Fig. 4B). To block cell-free transmission, as previously shown (14), extracellular infectious virus was neutralized by the addition of anti-E2 antibodies to the culture medium immediately following electroporation, as well as during the coculturing step. Effective blocking of cell-free infectivity was confirmed (Fig. S2A and B). Suppression of AP-1B and AP-4, but not AP-1A, significantly reduced HCV cell-to-cell spread (Fig. 4C). Overexpression of shRNA-resistant AP-1B and AP-4 (Fig. 3E) reversed the inhibitory effect of AP-1B and AP-4 depletion on cell-to-cell spread, respectively (Fig. 4D and E). Moreover, ectopic expression of AP-1A did not reverse the effect of AP-1B depletion on cell-to-cell spread, suggesting that the two adaptors do not have a redundant role in HCV spread. In contrast, ectopic expression of AP-1B in cells depleted of AP-1A increased cell-to-cell spread relative to the comparable level measured in AP-1A-depleted cells and NT controls expressing a control plasmid (Fig. 4D and F). In agreement with these results, ectopic expression of AP-1B and AP-4, but not AP-1A, in WT Huh7.5 cells increased cell-to-cell spread, further supporting the differential roles of these adaptors in viral spread (Fig. 4D and G). Whereas depletion of AP-1A, AP-1B, and AP-4 suppressed the release of HCV genotype 1a H77S (53), depletion of AP-1B and AP-4, but not AP-1A, suppressed cell-to-cell H77S spread (Fig. S2C to F), indicating that the observed differential adaptor requirement is not specific to genotype 2a.

10.1128/mBio.02233-17.3 FIG S2  Differential requirement for host APs in distinct modes of HCV spread. (A, B) Neutralizing anti-E2 antibodies inhibit cell-free infectivity. HCV RNA-transfected donor cells were cocultured with GFP-expressing target cells in the presence of neutralizing anti-E2 antibodies (CBH-5) or a human IgG isotype control (Thermo Fisher no. 12000C) at a concentration of 20 μg/ml. Supernatants derived from these cocultures were used to inoculate naive Huh7.5 cells; this was followed by staining of HCV NS5A and flow cytometry. (A) Representative FACS plots at 24 h postinoculation. (B) Quantitative infectivity data measured via flow cytometry at 24 and 48 h postinoculation. (C to F) HCV RNA replication at 5 and 72 h (C), intra- and extracellular infectivity at 72 h (D), and cell free (E) and cell-to-cell (F) spread in Huh7.5 cells electroporated with genotype 1a H77S luciferase reporter HCV. Results representative of at least two experiments conducted are shown. Shown are the mean ± SD. *, *P* < 0.05; **, *P* < 0.01; ***, *P* < 0.001 (relative to the corresponding IgG [B] or NT control [C to F]; one-way [D to F] or two-way [C] ANOVA with Dunnett’s *post hoc* test and two-tailed unpaired *t* test [B]). Download FIG S2, TIF file, 0.3 MB. Copyright © 2018 Xiao et al. 2018 Xiao et al. This content is distributed under the terms of the Creative Commons Attribution 4.0 International license.

Together, these data demonstrate a differential coopting of APs in distinct modes of viral spread and are in line with the functions of these AP adaptors in polarized epithelia (17, 20–23).

### HCV particles cotraffic with AP-4 in a post-TGN compartment.

To define the mechanism by which AP-4 mediates its role in HCV infection, we tested our hypothesis that AP-4 facilitates HCV particle trafficking during viral release. To do so, we used live-cell imaging to monitor the cotrafficking of individual, infectious HCV particles harboring a tetracysteine (TC) tag within the core protein (TC-core) with AP-4. We have previously shown that while static TC-core associates with LDs, motile TC-core requires HCV virion assembly (8, 54). Motile TC-core cotraffics with RNA, ApoE, microtubules, and components of the secretory pathway (8). Additionally, a fraction of HCV TC-core particles cotraffics with the AP-1A (25%) or AP-2 (38%) complexes (39).

**FIG 5:**
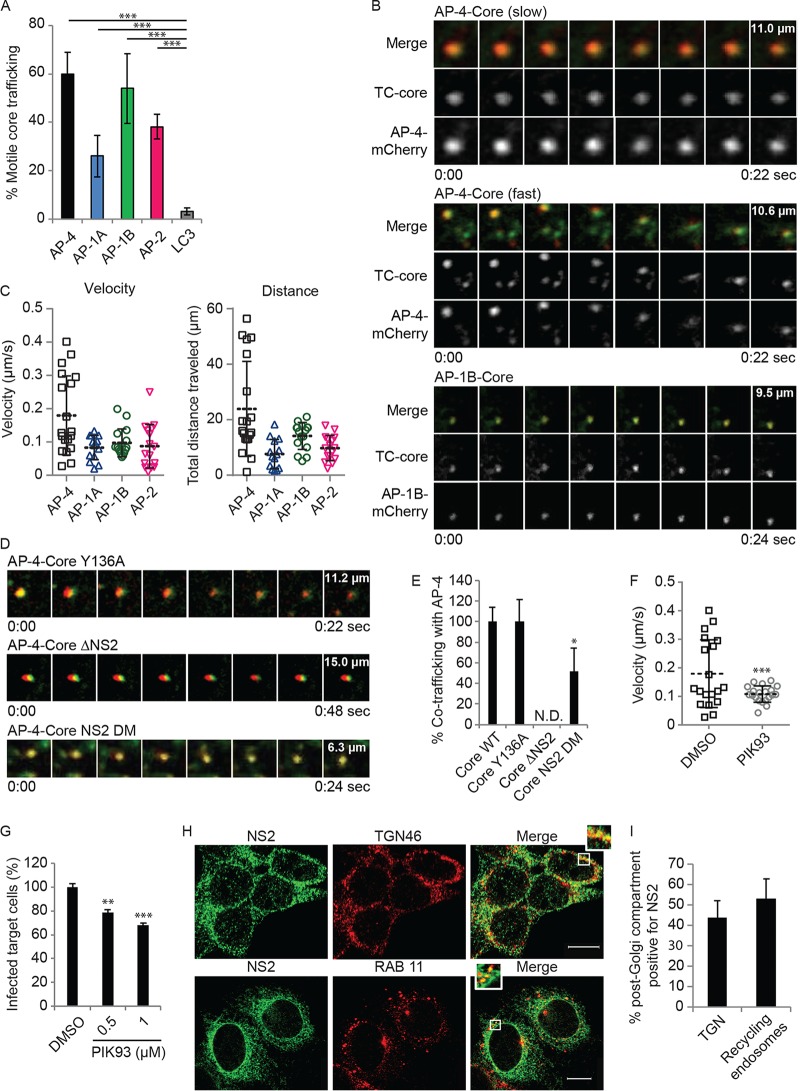
HCV particles cotraffic with AP-4 in a post-TGN compartment. (A) Quantification of motile TC-core puncta cotrafficking with AP-4, AP-1A, AP-1B, AP-2, and LC3. (B) Representative live-cell fluorescence microscopy montages of TC-core HCV (green) cotrafficking with AP-4-mCherry (top and panels middle) or AP-1B-meCherry (bottom) (red). The time elapsed (seconds) during video acquisition and the vertical dimension of the crop (micrometers) are indicated. (C) Velocity (left) and total distance traveled (right) of individual TC-core puncta cotrafficking with AP-4, AP-1A, AP-1B, or AP-2. (D and E) Representative montages (D) and quantitative data relative to WT TC-core (E) from live cell fluorescence microscopy of AP-4 cotrafficking with Y136A (top), NS2 deletion (middle), and NS2 double dileucine (DM; bottom) TC-core mutants. (F) Quantification of velocity per acquisition of WT TC-Core associated with AP-4 upon treatment with PIK93. (G) HCV cell-to-cell spread measured by FACS analysis following a 6-h treatment of cocultures of HCV RNA-transfected Huh7.5 donor cells and GFP-expressing target cells with PIK93. (H) Representative confocal IF microscopy images at ×40 magnification of NS2 (green) and TGN46 (red) or RAB11 (red) in HCV-transfected cells. *n* = >25. Scale bars represent 10 µm. (I) Quantitative colocalization analysis of *z* stacks by using Manders’ colocalization coefficients. Mean M2 values are represented as percent colocalization (the fraction of green intensity that coincides with red intensity ± SD). N.D., not detected. Experiments were replicated at least twice. *, *P* < 0.05; **, *P* < 0.01; ***, *P* < 0.001 (one-way ANOVA with Dunnett’s *post hoc* test [A, E, and G] or two-tailed unpaired *t* test [F]).

Analysis of TC-core puncta stained with the biarsenical dye FlAsH revealed that the largest fraction of motile TC-core cotrafficked with AP-4 (~60%), followed by AP-1B (~54%), whereas only 3% cotrafficked with the autophagosomal marker LC3 (Fig. 5A). Approximately half of the TC-core/AP-4 puncta move with velocities and distances similar to those of AP-1A, AP-1B, and AP-2 (Fig. 5B and C; Movies S1 to S3). These slower, short-range, AP-4-associated TC-core puncta traffic at velocities that are consistent with previous reports on secretory vesicle trafficking and TC-core punctum cotrafficking with vesicle-associated membrane protein (VAMP), AP-1A, and AP-2 (8, 39, 55) (Fig. 5B and C). The remaining TC-core/AP-4 puncta cotraffic at significantly higher velocities and over longer distances than the other AP-associated puncta, suggesting that they may have a distinct trafficking pattern (Fig. 5B and C).

10.1128/mBio.02233-17.5 MOVIE S1  Representative video of cotrafficking of TC-core HCV with AP-4 (population 1). TC-core is green, and AP-4 is red. Download MOVIE S1, AVI file, 0.3 MB. Copyright © 2018 Xiao et al. 2018 Xiao et al. This content is distributed under the terms of the Creative Commons Attribution 4.0 International license.

10.1128/mBio.02233-17.6 MOVIE S2  Representative video of cotrafficking of TC-core HCV with AP-4 (population 2). TC-core is green, and AP-4 is red. Download MOVIE S2, AVI file, 0.3 MB. Copyright © 2018 Xiao et al. 2018 Xiao et al. This content is distributed under the terms of the Creative Commons Attribution 4.0 International license.

10.1128/mBio.02233-17.7 MOVIE S3  Representative video of cotrafficking of TC-core HCV with AP-1B. TC-core is green, and AP-1B is red. Download MOVIE S3, AVI file, 0.01 MB. Copyright © 2018 Xiao et al. 2018 Xiao et al. This content is distributed under the terms of the Creative Commons Attribution 4.0 International license.

Next, we determined the role of core and NS2, two AP binding viral proteins required for virion assembly, in HCV particle trafficking with AP-4. To do so, we monitored the cotrafficking of AP-4 with TC-core HCV harboring either a mutation (Y136A) within a YXXØ motif of core, a motif critical for interaction and cotrafficking with AP-2 and for HCV assembly (36, 39), or a deletion of NS2. The Y136A core mutation did not alter AP-4 colocalization with TC-core puncta (Fig. 5D; Movie S4). Moreover, while this mutation reduced the overall percentage of motile TC-core puncta by >60% relative to WT TC-core, it did not alter the proportion or velocity of particles that cotraffic with AP-4 (Fig. 5D and E; Movie S4). TC-core puncta only partially overlapped AP-4 puncta in the context of infection with the NS2 deletion virus, suggesting that they are juxtaposed (Fig. 5D and E). However, AP-4-associated TC-core punctum motility was entirely abolished (0% moving particles) upon the deletion of NS2, as measured by the overall distance traveled (Fig. 5D and E; Movie S5). These results suggest that while another viral protein may be required to recruit AP-4 to the proximity of HCV sites of assembly, NS2 is absolutely essential for full colocalization of HCV particles with AP-4 and their cotrafficking. Since NS2 is essential for virion assembly, we introduced the AP-4 binding dileucine motif double mutation into TC-core (TC-core DM). Cotrafficking of this TC-core DM with AP-4 was significantly reduced relative to that of WT TC-core, although some cotrafficking occurred with the mutant (Fig. 5D and E; Movie S6). This suggests that the dileucine motifs are required for some AP-4 cotrafficking (and functional release) and that either a subset of cotrafficking is independent of the dileucine motifs or, alternatively, the mutation reduces but does not abolish AP-4 interactions (as in Fig. 2C).

10.1128/mBio.02233-17.8 MOVIE S4  Representative video of cotrafficking of TC-core Y136A HCV with AP-4. Mutant TC-core is green, and AP-4 is red. Download MOVIE S4, AVI file, 0.3 MB. Copyright © 2018 Xiao et al. 2018 Xiao et al. This content is distributed under the terms of the Creative Commons Attribution 4.0 International license.

10.1128/mBio.02233-17.9 MOVIE S5  Representative video of cotrafficking of NS2 deleted TC-core HCV with AP-4. Mutant TC-core is green, and AP-4 is red. Download MOVIE S5, AVI file, 1 MB. Copyright © 2018 Xiao et al. 2018 Xiao et al. This content is distributed under the terms of the Creative Commons Attribution 4.0 International license.

10.1128/mBio.02233-17.10 MOVIE S6  Representative video of cotrafficking of TC-core HCV harboring the dileucine DM with AP-4. Mutant TC-core is green, and AP-4 is red. Download MOVIE S6, AVI file, 0.01 MB. Copyright © 2018 Xiao et al. 2018 Xiao et al. This content is distributed under the terms of the Creative Commons Attribution 4.0 International license.

Phosphatidylinositol-4 kinase IIIβ is a Golgi compartment lipid kinase that is important in Golgi compartment structure and function, in addition to being involved in HCV egress (8, 56). PIK93 is a kinase inhibitor that at 0.5 μM inhibits phosphatidylinositol-4 kinase IIIβ (57, 58). This concentration of PIK93 inhibits TC-core trafficking to post-Golgi compartments, leading to enhanced accumulation in the TGN (8). To test our hypothesis that cotrafficking of viral particles with AP-4 is via the secretory pathway, we treated HCV-infected cells with 0.5 μM PIK93. PIK93 treatment did not alter colocalization of AP-4 with TC-core puncta or the traffic of slowly moving TC-core–AP-4 puncta; however, it eliminated the rapidly moving, long-range TC-core–AP-4 puncta (Fig. 5F). PIK93 thus significantly reduced the average velocity of the population of rapidly moving TC-core puncta cotrafficking with AP-4. The rapidly moving particles that cotraffic with AP-4 are therefore indicative of transport at a post-Golgi compartment. Moreover, a 6-h PIK93 treatment of cocultures of HCV RNA-transfected and GFP target cells inhibited cell-to-cell spread (Fig. 5G), suggesting that the cotrafficking of the rapidly moving particles with AP-4 is involved in cell-to-cell spread. To further define the role of NS2 in the observed transport, we studied its localization in post-Golgi compartments by a quantitative confocal IF analysis. As shown in Fig. 5H and I, 44% ± 8% of the TG46-labeled TGN and 53% ± 10% of the RAB11-labeled recycling endosomes stained positive for NS2 in HCV-infected cells (*n* = 25 to 40 cells), suggesting localization in post-Golgi compartments in addition to the ER and ER-derived structures.

Together, these findings reveal the mechanistic involvement of AP-4 in late steps of the HCV life cycle and provide direct evidence of a role for additional APs in the mediation of intracellular viral trafficking.

### AP-1B regulation by AAK1 and GAK modulates NS2 binding and HCV cell-to-cell spread.

**FIG 6:**
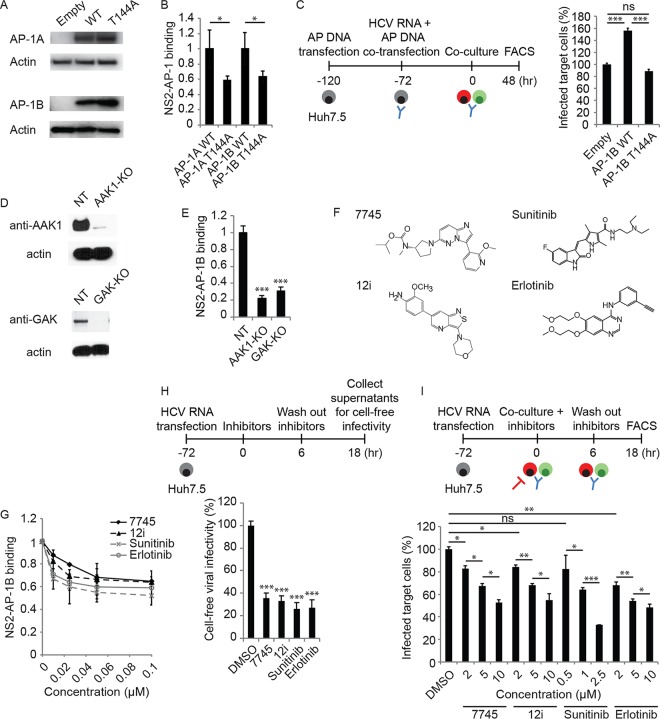
AAK1 and GAK regulate NS2–AP-1 binding and HCV cell-free and cell-to-cell spread. (A) AP-1 expression following transfection of Huh7.5 cells with GLuc-tagged WT or T144A mutant AP-1A/B or an empty control and blotting with anti-GLuc and anti-actin antibodies. (B) NS2 binding to WT and T144A mutant AP-1A/B measured by PCA. Data are plotted relative to the respective WT control. (C) HCV cell-to-cell spread in AP-1B-overexpressing cells versus an empty-vector control measured via FACS analysis 48 h following coculturing of HCV RNA-transfected donor Huh7.5 cells with GFP-expressing target cells. (D) Confirmation of gene expression knockout (KO) by Western blotting in Huh7.5 cells transduced with CRISPR subgenomic RNA lentivirus targeting AAK1 or GAK or an NT control. (E) NS2 binding to AP-1B measured in AAK1 and GAK knockout cells by PCAs. Data are plotted relative to the control cells. (F) Chemical structures of the compounds indicated. (G) Effects of the compounds indicated on NS2–AP-1B binding measured by PCA. (H) Cell-free infectivity of culture supernatants collected following a 6-h treatment of HCV RNA-transfected cells with the four individual compounds at a concentration of 10 μM (compound 7745, 12i, and erlotinib) or 2.5 μM (sunitinib), followed by compound removal and a 12-h incubation in fresh medium, measured via luciferase assay at 72 h postinoculation of naive cells. (I) Dose response of HCV cell-to-cell spread to the compounds indicated measured by FACS analysis following a 6-h treatment of cocultures of HCV RNA-transfected Huh7.5 donor cells and GFP-expressing target cells. Shown in panels B, C, E, and G to I are representative results of experiments from at least two conducted, each with three to six biological replicates. Shown are the mean ± SD. ns, not significant; *, *P* < 0.05; **, *P* < 0.01; ***, *P* < 0.001 (relative to WT AP-1 [B] or an empty-vector [C], NT [E], or vehicle [G to I] control; two-tailed unpaired *t* test [B], one-way ANOVA with Tukey’s [C] or Dunnett’s [E, H, and I] *post hoc* test).

Next, we tested whether AAK1 and GAK regulate NS2–AP-1 binding by studying the effect of phosphorylation site (T144A) mutant AP-1 (59) on NS2 binding. A T144A mutation in AP-1A or AP-1B did not impair protein expression, yet it reduced NS2 binding by ~2-fold, as measured by PCAs (Fig. 6A and B).

We previously reported that ectopic expression of WT but not T144A mutant AP-1A increased HCV release (39). Here, we studied the role of the T144 residue of AP-1B in the regulation of cell-to-cell spread by using coculture assays as described above. Ectopic expression of WT but not T144A AP-1B mutant increased HCV cell-to-cell spread (Fig. 6C). Moreover, in contrast to WT AP-1B (Fig. 4E), overexpression of T144A mutant AP-1B did not reverse the defect in cell-to-cell spread induced by AP-1B suppression (data not shown).

To test our hypothesis that AAK1 and GAK regulate the NS2–AP-1B interaction, we conducted binding experiments with Huh7.5 cells following the knockout of AAK1 or GAK by CRISPR/Cas9 (Fig. 6D). Knockout of either AAK1 or GAK significantly reduced NS2–AP-1B binding compared to that seen with an NT control, as measured by PCAs (Fig. 6E).

These results suggest that AP-1A and AP-1B phosphorylation by AAK1 and GAK stimulates their binding to NS2 and regulates AP-1B-mediated HCV cell-to-cell spread in addition to cell-free viral release (39).

### Pharmacological inhibition of NS2–AP-1B binding and HCV cell-to-cell spread.

To determine whether NS2–AP-1B binding and HCV cell-to-cell spread could be inhibited pharmacologically and further validate AAK1 and GAK as antiviral targets, we treated cells with selective AAK1 and GAK inhibitors. Compound 7745 is an imidazo[1,2-b]pyridazine-based molecule originally developed to modulate AAK1 activity as a potential treatment of neurological disorders (*K*_*d*_ = 1 nM; 50% inhibitory concentration, <10 nM) (Fig. 6F) (39, 60). The isothiazolo[5,4-b]pyridine 12i (Fig. 6F) is a potent (*K*_*d*_ = ~8 nM), selective, ATP-competitive GAK inhibitor capable of restricting HCV and dengue virus (DENV) infections (39, 41). We measured the inhibition of NS2–AP-1B binding following treatment with both compounds (Fig. 6G). Consistent with these findings, we also measured the inhibition of NS2–AP-1B binding by PCAs upon treatment with sunitinib and erlotinib, two approved anticancer drugs with potent, albeit nonselective, anti-AAK1 and/or anti-GAK activity (Fig. 6G).

We previously reported that these compounds inhibit the entry and intra- and extracellular infectivity of HCV and DENV (35, 36, 39, 41). Here, we used these pharmacological tools to probe the role of AAK1 and GAK in HCV cell-free and cell-to-cell spread independently of their roles in viral entry and assembly. To measure the effect of these compounds on cell-free infectivity, HCV RNA-transfected Huh7.5 cells were treated at 72 h posttransfection for 6 h (Fig. 6H). Compounds were then washed, cells were incubated with fresh medium for an additional 6 or 12 h, and supernatants were used to inoculate naive cells, which were then subjected to luciferase assays at 72 h. Treatment with the individual four compounds for 6 h (Fig. S3A) or 12 h (Fig. 6H) significantly reduced cell-free infectivity. The observed defect in HCV cell-free infectivity was not associated with increased release of noninfectious viral particles, as indicated by the levels of HCV RNA and core protein in supernatants derived from cells depleted of these APs (Fig. S3B and C).

10.1128/mBio.02233-17.4 FIG S3  Pharmacological inhibition of HCV cell-free and cell-to-cell spread. (A) Cell-free infectivity of culture supernatants collected following a 6-h treatment of HCV RNA-transfected cells with the individual compounds at a concentration of 10 μM, followed by compound removal and a 6-h incubation in fresh medium, measured via luciferase assay at 72 h postinoculation of naive cells. (B and C) Viral RNA (B) and core protein (C) release into the culture supernatant measured at 12 h after a 6-h treatment with the compounds indicated and their washout measured by qRT-PCR and ELISA, respectively. (D) Intracellular infectivity measured via luciferase assay in naive cells inoculated with lysates derived from HCV RNA-electroporated cells following a 6-h treatment with the compounds indicated at a concentration of 10 μM (compound 7745, 12i, and erlotinib) or 2.5 μM (sunitinib). Treatment was initiated at 72 h postelectroporation. Results represent data pooled from at least two independent experiments each with 3 to 10 biological replicates. Shown are the mean ± SD. *, *P* < 0.05; **, *P* < 0.01; ***, *P* < 0.001 (relative to a DMSO control; one-way ANOVA with Dunnett’s *post hoc* test). Download FIG S3, TIF file, 0.1 MB. Copyright © 2018 Xiao et al. 2018 Xiao et al. This content is distributed under the terms of the Creative Commons Attribution 4.0 International license.

To determine an effect on cell-to-cell HCV spread, treatment was initiated upon the coculturing of Huh7.5 HCV donor cells and GFP-expressing target cells for 6 h in the presence of neutralizing anti-E2 antibodies (Fig. 6I). Following the removal of residual inhibitors and 12 h of incubation, cocultures were stained with anti-NS5A antibody and analyzed by flow cytometry. Treatment with various concentrations of all four compounds resulted in dose-dependent inhibition of cell-to-cell spread (Fig. 6I), with no appreciable toxicity at the concentrations used (data not shown). Notably, a 6-h treatment of HCV RNA-transfected cells with the four individual compounds had no effect on the assembly of new viral particles, as measured by intracellular infectivity (Fig. S3D). These findings indicate that the effect of these compounds on HCV cell-free and cell-to-cell spread is independent of their effect on HCV entry and assembly.

Together, these data suggest that, in addition to regulating HCV entry and assembly (35, 36), AAK1 and GAK are involved in the regulation of HCV cell-free and cell-to-cell spread.

## DISCUSSION

It has been unknown which viral determinants and cellular adaptors are essential for HCV particle traffic during the release of cell-free- or cell-to-cell-transmitted virus. And while clathrin-associated AP-1 and AP-2 complexes have been implicated in multiple viral infections (31, 32, 61, 62), the involvement of the non-clathrin-associated AP-4 complex in viral infection and its precise mechanistic involvement were not characterized. Moreover, the relevance of AP complexes to viral cell-to-cell spread remained unknown. Here, we set out to address this knowledge gap. By integrating proteomic, RNA interference (RNAi), viral genetic, advanced live-cell imaging, and pharmacological approaches, we provide evidence that two conserved heretofore unrecognized dileucine motifs in NS2 mediate AP-1A, AP-1B, and AP-4 binding and HCV release. Moreover, we demonstrate differential HCV hijacking of these AP complexes to facilitate the trafficking of HCV particles during cell-free and cell-to-cell spread. Lastly, we establish that, in addition to viral entry, assembly, and cell-free virus release, HCV cell-to-cell spread is also regulated by AAK1 and GAK and is susceptible to their pharmacological inhibition.

NS2 was identified in our PCA screening and co-IP experiments as a prominent binding partner of the μ subunits of AP-1A, AP-1B, and AP-4. AP-4 also interacted with NS5A. These data, together with the finding that AP-2 binds HCV core with the highest apparent affinity, indicate differential binding of APs by individual HCV proteins. Moreover, we reveal that two dileucine motifs within NS2 mediate AP-1A, AP-1B, and AP-4 binding. These results are in agreement with prior reports of tyrosine and dileucine motif binding to AP μ subunits (31–34) and display the flexibility of interactions for an individual AP binding motif with the μ subunits of several distinct AP complexes (31, 63, 64).

Infectivity assays revealed that, in addition to previously reported N-terminal residues (11), the dileucine motifs in the C terminus of NS2 are also essential for HCV release. Whereas NS2 mutations had no effect on the absolute intracellular infectivity, they significantly increased the ratio of intracellular infectivity to total (intra- plus extracellular) infectivity in line with prior reports (11). The effect of NS2 mutations on HCV release was greater than their effect on AP binding, presumably because of differences in the dynamic range of the assays. Nevertheless, our data indicate that these motifs are required for a functional interaction. It was previously speculated that the L217 residue, which is locked in the active site of the protease domain of NS2, mediates its role in infectious virus production via interactions with host proteins (9). We provide evidence that L217 and additional NS2 C-terminal leucine residues play a role in infectious HCV production in part by binding AP-1A, AP-1B, and AP-4. Some of the leucine residues are within the NS2 dimer interface based on the structure of the catalytic domain of the NS2-3 protease (46). Nevertheless, it is possible that the interaction with APs is via the monomeric and not the dimeric form of NS2, suggesting a potential change in confirmation, as previously shown with other viral (65) and host (66) proteins. While the first leucine motif includes a functional acidic residue (D207), the second motif resembles previously described dileucine-based motifs lacking acidic residues (67, 68). We thereby define the mechanism by which NS2 interacts with host APs and genetically validate the requirement for two dileucine motifs in HCV release.

We previously reported that AP-2 is essential for HCV assembly (36). Moreover, we and others showed that AP-1A is required for HCV release (8, 37–39). In this study, we extended these observations by demonstrating a role for AP-1B in HCV infection and for AP-4 in any viral infection. Moreover, prior studies have focused on the mechanisms that facilitate HCV entry into the recipient neighboring cell (12, 51, 69), rather than those involved in the sorting of virions toward the sites of cell-cell contact in the plasma membrane within the donor cell. Our data thus provide insight into these mechanisms. Since cell-to-cell spread represents a mechanism by which HCV evades immune clearance and establishes persistence (12–14), our results suggest that host APs may be involved in the facilitation of HCV persistence. Notably, a tyrosine-based motif in the HIV-1 envelope glycoprotein is essential for the mediation of cell-to-cell spread (70), yet a requirement for APs in the cell-to-cell spread of a virus has not been described yet. Our findings may thus encourage future investigations of these mechanisms in other viruses that establish persistent infection.

Our advanced live-cell imaging data provide direct evidence that the majority of HCV particles cotraffic intracellularly with AP-4, followed by AP-1B, AP-1A, and AP-2, thereby indicating the critical roles of AP-4 in intracellular HCV traffic. These results raise the following question: why does HCV utilize three adaptors to traffic during its release? We favor the explanation that AP-1A, AP-1B, and AP-4 mediate viral traffic in distinct pathways. Although all three complexes sort in post-Golgi compartments, multiple studies indicate that they function in physically and functionally distinct membrane domains. AP-1B and AP-4 have been implicated in sorting to the basolateral membrane, while AP-1A and AP-4 have been implicated in sorting between the TGN and recycling endosomes (20, 21). Whereas there is evidence that AP-1A can also be involved in basolateral sorting and that AP-1B can compensate for a lack of AP-1A, this was documented specifically in MDCK cells (71), whose protein trafficking is substantially different from that of hepatocytes (72).

Differences in the μ subunits of the otherwise closely homologous AP-1A and AP-1B complexes (79% amino acid sequence identity [73]) and differential preferences for specific membrane lipids define their distinct functions (20, 22, 74, 75). Our data provide evidence that these two distinct proteins have differential functionality in HCV release, yet their patterns of cotrafficking with TC-core in terms of distance and velocity are similar. This suggests that AP-1A and AP-1B mediate distinct trafficking pathways that operate with similar kinetics. Indeed, our finding that ectopic expression of AP-1A does not reverse the effect of AP-1B depletion on cell-to-cell spread suggests that the two adaptors do not have a redundant role in HCV spread and are in line with prior reports that AP-1A cannot substitute for AP-1B in the basolateral sorting of cellular receptors (76, 77).

We demonstrate that while AP-1A, AP-1B, and AP-2 cotraffic with TC-core particles over short distances, two patterns of movement characterize HCV particles that cotraffic with AP-4: a slow, short-range pattern and a fast, long-range pattern. The short-range moving population that cotraffics with AP-1A, AP-1B, and AP-4 is consistent with the previously reported TC-core particles associated with apolipoprotein E and VAMP1 vesicles (8). On the basis of the functions of these APs in polarized epithelia, we predict that these particles traffic from the TGN to recycling endosomes. Since pharmacological inhibition of trafficking from the Golgi compartment to the plasma membrane disrupts both AP-4-associated long-range movement and HCV cell-to-cell spread and since AP-4 mediates cell-to-cell spread, the long-range pattern likely represents basolateral sorting. Our data also demonstrate that even though both AP-1B and AP-4 mediate cell-to-cell spread, the patterns of their cotrafficking with TC-core are different. This suggests that the two adaptors may either mediate trafficking in distinct pathways or act in two steps in the same pathway. Interestingly, in contrast to the clathrin-associated AP-1A, AP-1B, and AP-2 complexes, AP-4’s coat protein has not been identified (19), and this complex mediates cargo transport in a clathrin-independent manner (78), providing a possible explanation for the observed differential phenotype. Together, our imaging findings contribute to our understanding of the complex mechanism by which these APs are involved in infectious HCV production and exclude a theory whereby AP complexes contribute to viral infections solely by recruiting or mediating intracellular traffic of host cargo components essential for the viral life cycle or by mediating other roles (e.g., HCV E2 stabilization [38]). Further investigation is, however, required to understand exactly where in the secretory pathway these APs exert their functions. Although these APs appear to maintain at least some of their distinct sorting properties in our nonpolarized Huh7.5 cell culture model, it will be important to validate these findings in a more biologically relevant polarized cell model that supports authentic apical and basolateral sorting events. It is also possible that by interacting with several complexes, HCV coopts differential functions of these APs beyond post-Golgi traffic, such as exocyst complex recruitment for fusion with the plasma membrane by AP-1B (20). Lastly, utilizing different adaptors could facilitate differential and/or tighter regulation of HCV traffic.

We previously showed that AAK1 and GAK regulate HCV assembly and release by phosphorylating AP-2 and AP-1A, respectively (36, 39). Here, we show that regulation of AP-1B T144 by AAK1 and GAK increases both its apparent affinity for NS2 and cell-to-cell spread. Moreover, using pharmacological inhibitors of AAK1 and GAK, we further establish a novel role for these kinases in the regulation of cell-free and cell-to-cell viral spread. Since treatment with the kinase inhibitors was limited to 6 to 12 h (an incubation time that we show is too short to suppress viral assembly), the observed impairment of viral cell-free and cell-to-cell spread was likely to have resulted not from suppressed viral assembly but rather from suppressed spread of preformed particles. Additionally, collection of supernatants 12 h following compound removal from cell culture and inclusion of neutralizing anti-E2 antibodies enabled measurements of these compounds’ effect on cell-free and cell-to-cell spread, respectively, independently of their effect on viral entry. These findings underscore the utility of these pharmacological tools in probing distinct steps of the HCV life cycle. Furthermore, they point to the potential of this antiviral approach to suppress the establishment and/or maintenance of viral persistence and possibly reduce viral escape and treatment failure (15, 16). Higher concentrations of the compounds were required to inhibit HCV release and cell-to-cell spread than to inhibit NS2–AP-1 binding. Such differences between biological and biochemical effects are typical for kinase inhibitors and often result from limited permeability and/or efflux (36, 79).

**FIG 7:**
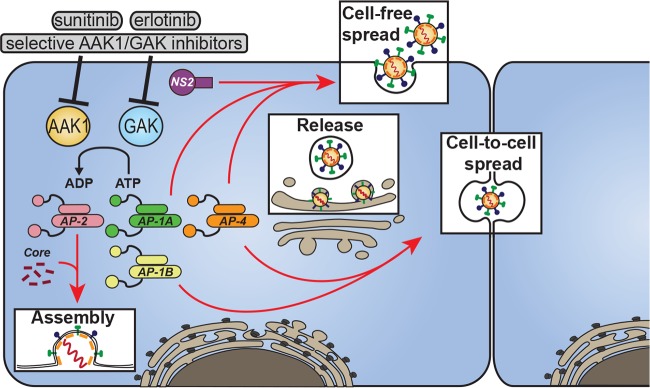
Model showing the orchestration of viral trafficking in late steps of the HCV life cycle by AP complexes and their regulators. HCV hijacks the AP-1A, AP-1B, AP-2, and AP-4 complexes to mediate intracellular traffic of viral particles in temporally distinct late steps of its life cycle. Host kinases AAK1 and GAK regulate the assembly and release of cell-free- and cell-to-cell-transmitted virus through phosphorylation of AP-1 (green and yellow) and AP-2 (pink). Sunitinib, erlotinib, and selective inhibitors of AAK1 and GAK disrupt these temporally distinct steps of the viral life cycle.

On the basis of these mechanistic data and our former work (35, 36, 39, 41), we propose a model wherein HCV proteins bind AP complexes to mediate intracellular traffic of HCV particles in temporally distinct steps of infectious HCV production (Fig. 7). In addition to playing an essential role in HCV endocytosis, AP-2 is recruited to the surface of LD by a tyrosine motif within core to mediate a role in HCV assembly (36). AP-1A, AP-1B, and AP-4 are recognized by dileucine motifs within NS2, and these interactions mediate HCV release. AP-1B and AP-4 also play a role in HCV cell-to-cell spread. The host cell kinases AAK1 and GAK represent “master regulators” of HCV infection; by stimulating AP-1A, AP-1B, and AP-2 interactions with host and viral proteins (such as core and NS2), they regulate HCV entry, assembly, release of cell-free virus, and cell-to-cell spread. Kinase inhibitors with activity against AAK1 and GAK, including selective inhibitors and already approved nonselective drugs such as sunitinib and erlotinib, inhibit these temporally distinct steps of the viral life cycle, in part by suppressing the cotrafficking of HCV particles with AP-1A and AP-2 (39).

It remains to be investigated precisely how NS2-AP binding facilitates HCV particle traffic. In the case of HIV, the envelope protein is thought to interact with APs to mediate traffic (34, 64). Similarly, HCV E2 was recently reported to bind AP1S3, the σ subunit of the AP-1 complex (38). However, it is unclear how this interaction can occur topologically, since in contrast to HIV Env, the HCV E1 and E2 glycoproteins lack a cytosolic domain. NS2 was previously reported to mediate HCV release (11) and to alter the TGN architecture when expressed in the context of the HCV replicase (40), suggesting that in addition to the ER, it is localized in post-Golgi compartments. Indeed, our confocal IF analysis demonstrated significant localization of NS2 in both the TGN and recycling endosomes. One model predicts that NS2 may recognize a lipid enriched in HCV-containing vesicles, thereby bridging between the luminal virions and the cytoplasmic APs. Alternatively, a transmembrane protein or protein complex may bind NS2 and a component of the virion. NS2 would then bind the AP complex, which recruits clathrin in the case of AP-1A and AP-1B (and another coat in the case of AP-4). Notably, our cotrafficking data indicate that upon NS2 deletion, TC-core puncta and AP-4 are not fully colocalized but rather juxtaposed, and the motility of TC-core punctum cotrafficking with AP-4 is completely abolished. These data support a critical role for NS2 in facilitating cotraffic with AP-4 but also suggest the existence of another factor(s) for the recruitment of AP-4 to the vicinity of HCV sites of assembly. Intriguingly, NS5A was identified in our PCA screening as another potential partner of AP-1A, AP-1B, and AP-4. We thus speculate that these complexes are first recruited, possibly by NS5A, and subsequently bind NS2. By interacting with the envelope glycoproteins within the secretory vesicle and the cytoplasmic AP complex, the transmembrane NS2 facilitates the traffic of viral particles between various distinct compartments in the secretory pathway.

In summary, our study uncovers novel virus and host determinants, as well as molecular mechanisms, underlying differential HCV trafficking during the release of cell-free- and cell-to-cell-transmitted viral particles with potential implications for pathogenesis of viral persistence and the design of novel antiviral strategies.

## MATERIALS AND METHODS

The plasmids, reagents, antibodies, and RNAi used in this study are summarized in Text S1.

10.1128/mBio.02233-17.1 TEXT S1  Supplemental materials and methods and uncut versions of gels. Download TEXT S1, PDF file, 1.7 MB. Copyright © 2018 Xiao et al. 2018 Xiao et al. This content is distributed under the terms of the Creative Commons Attribution 4.0 International license.

### Cell cultures.

Huh7.5 (Apath LLC) and 293T (ATCC) cells were grown in Dulbecco’s modified Eagle’s medium supplemented with 10% fetal bovine serum (Omega Scientific), 1× nonessential amino acids, 1% l-glutamine, and 1% penicillin-streptomycin (Gibco).

### PCAs.

PCAs with mammalian cells were conducted as described previously (36, 42) and in Text S1.

### Co-IPs.

Co-IPs in membrane fractions derived from HCV RNA-transfected cells or cells ectopically expressing NS2 and AP-4 were carried out as described previously (36, 42) and in Text S1.

### *In vitro* transcription of HCV RNA, electroporation, and viral titration.

*In vitro* transcription of HCV RNA, electroporation, and viral titration were performed as previously reported (36).

### HCV RNA replication and intra- and extracellular infectivity.

As previously described (36, 50), HCV RNA replication was measured by luciferase assays (Promega) in Huh7.5 cell lysates 5 to 8 h and 72 h postelectroporation with J6/JFH(p7-Rluc2A) or pH77S.3/GLuc2A HCV RNA harboring a luciferase reporter. At 72 h, electroporated cells were trypsinized, centrifuged, resuspended in 500 µl of medium, lysed, and pelleted. To measure intra- and extracellular infectivity, these clarified lysates and culture supernatants, respectively, were used to inoculate naive cells in triplicate, followed by luciferase assays and limiting-dilution assays and 50% tissue culture infective dose (TCID_50_) calculation (44) at 72 h. Accumulation of infectious intracellular viral particles was calculated by dividing the intracellular infectivity by the sum of the intra- and extracellular infectivity for the dish.

### Cell-free infectivity.

As previously described (16, 51), Huh7.5 cells were electroporated with J6/JFH(p7-Rluc2A) RNA and incubated for 72 h. Culture supernatants were used to inoculate naive cells in triplicate, followed by luciferase assays at 6 h. When relevant, inhibitors were added at 72 h posttransfection for 6 h of incubation. Compounds were then washed, cells were incubated with fresh medium for an additional 6 or 12 h, supernatants were used to inoculate naive cells, and luciferase assays were performed at 72 h.

### Cell-to-cell spread assays.

As previously described (14, 16, 80), Huh7.5 cells were transfected with Jc1 (52) or H77S (53) RNA and cocultured with naive target Huh7.5-GFP cells at a 1:2 ratio in the presence of 2 µg/ml neutralizing human anti-E2 antibodies (CBH-5) to block the spread of cell-free virus. Samples were fixed and permeabilized by Cytofix/Cytoperm (BD), stained with mouse anti-NS5A antibodies (Virostat), and analyzed by flow cytometry (Stanford shared fluorescence-activated cell sorter [FACS] facility) at different time points following coculture. Cell-to-cell spread was defined as the percentage of newly infected target cells (GFP^+^ NS5A^+^) in the target cell population (GFP^+^).

### Viability assays.

Viability was assessed by using alamarBlue reagent (Invitrogen) in accordance with the manufacturer’s protocol. Fluorescence was detected at 560 nm on a Tecan Infinite M1000 reader.

### Core protein ELISA.

The concentration of released core protein was measured in clarified cell culture supernatants by ELISA (Cell Biolabs) against standard curves of recombinant core antigen in accordance with the manufacturer’s instructions.

### IF confocal microscopy.

IF confocal microscopy was performed with Huh7.5 cells at 72 h posttransfection with HCV RNA. Colocalization was quantified via ImageJ (JACoP) software and M2 Manders’ colocalization coefficients as previously described (36).

### Live-cell imaging.

Huh7.5 cells were infected with concentrated HCV TC-core at a multiplicity of infection of 1 for 24 h, transfected with AP-4–mCherry by using Lipofectamine 2000 (Invitrogen), and then seeded onto collagen-coated 35-mm FluoroDishes (World Precision Instruments, Inc.) (8, 39). When specified, TC-core-infected cells were incubated with dimethyl sulfoxide (DMSO) or PIK93 (0.5 µM) for 3 h beginning at 72 h postinfection. At 72 h postinfection, cells were labeled with biarsenical dye and time-lapse images were taken and analyzed as described in Text S1.

Generation of AAK1 and GAK knockout cell lines was performed as described in Text S1.

### Statistical analysis.

*P* values were calculated by two-tailed unpaired *t* test or one- or two-way analysis of variance (ANOVA) with either Dunnett’s or Tukey’s *post hoc* test.
