# Supplementary figures and images for: Interactions between the Hepatitis C Virus Nonstructural 2 Protein and Host Adaptor Proteins 1 and 4 Orchestrate Virus Release
Source: mBio. 2018 Mar 13;9(2):e02233-17. doi: 10.1128/mBio.02233-17 (PMC5850324; doi:10.1128/mBio.02233-17)

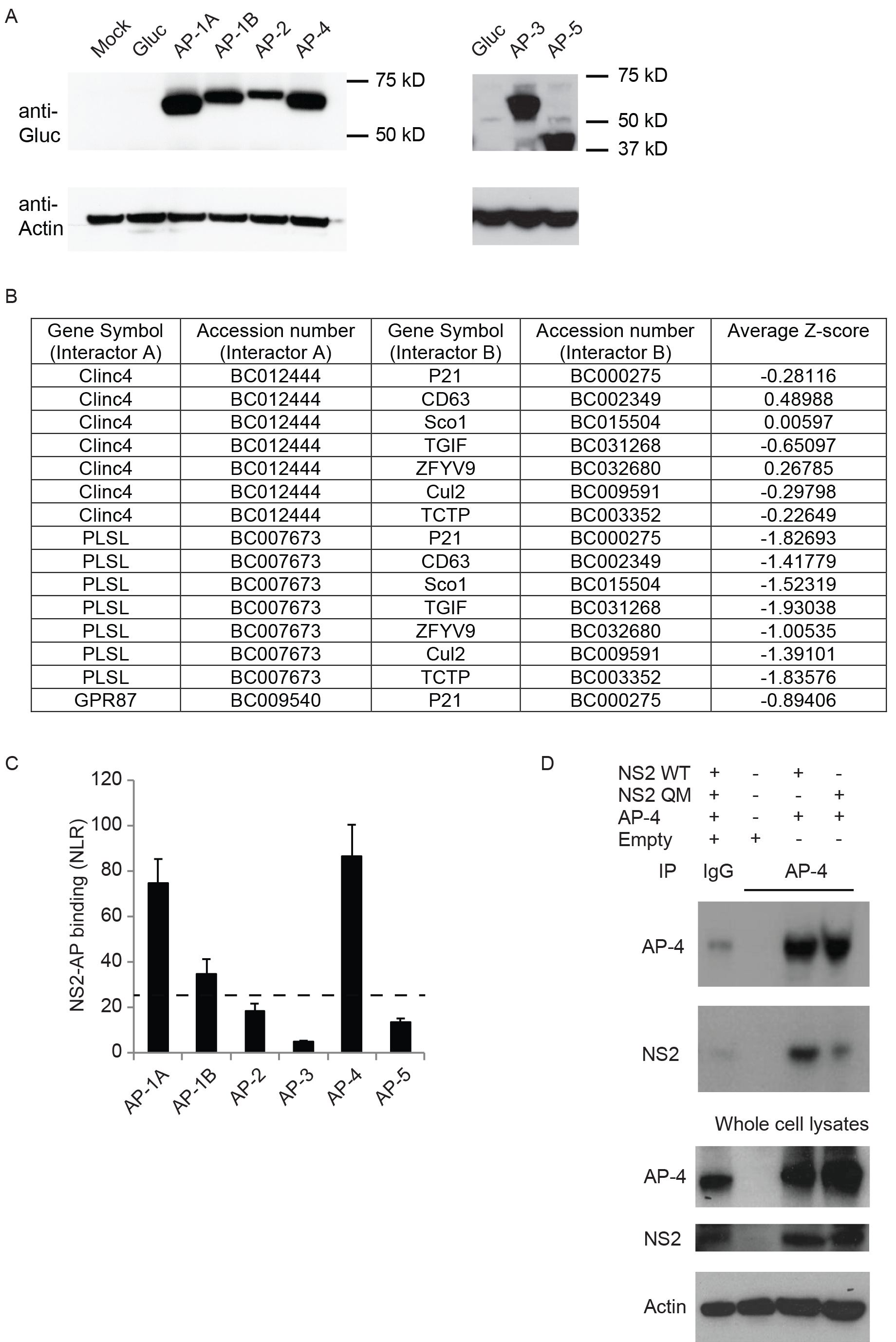

Supplement: FIG S1 [file mbo002183778sf1.tif]

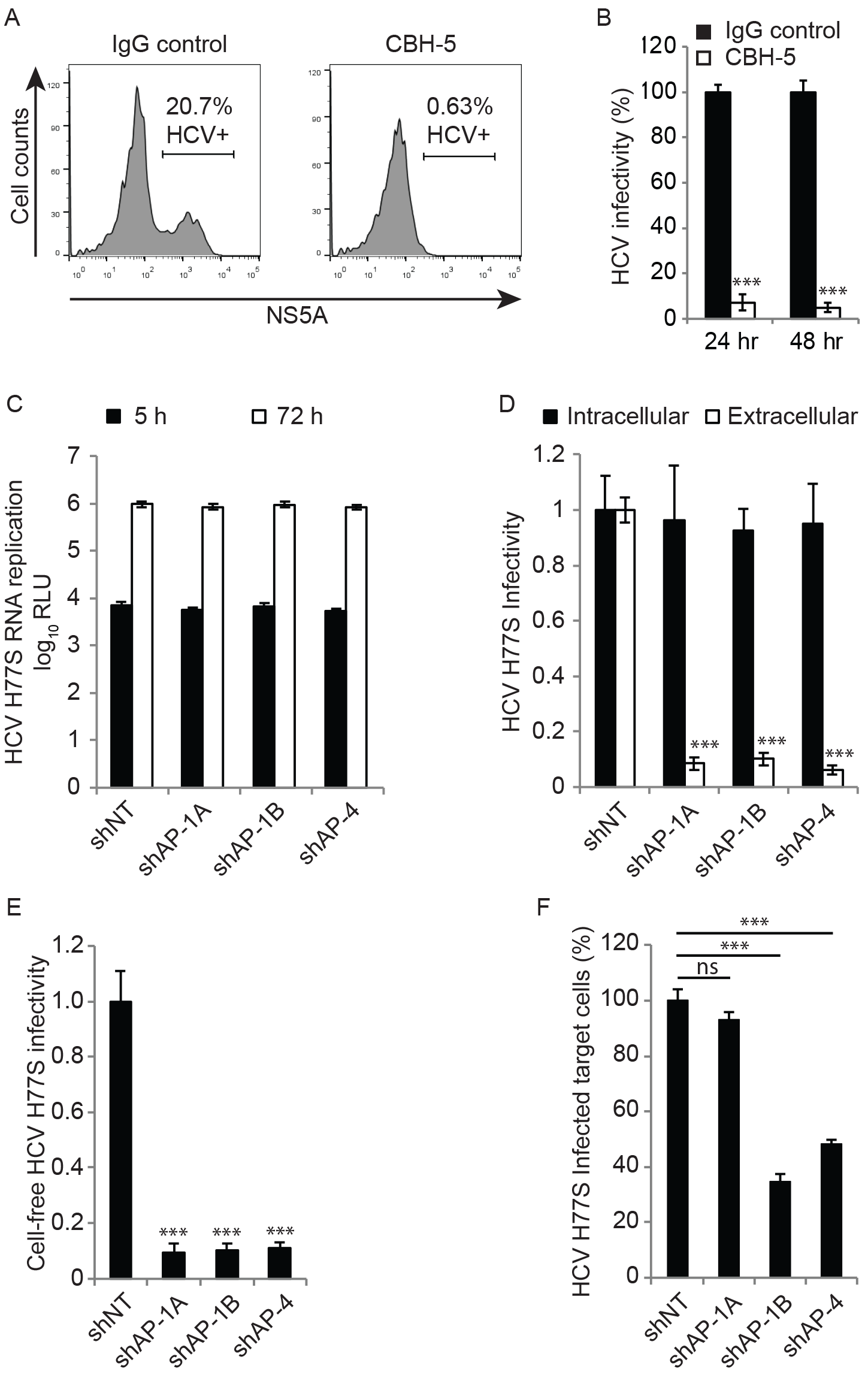

Supplement: FIG S2 [file mbo002183778sf2.tif]

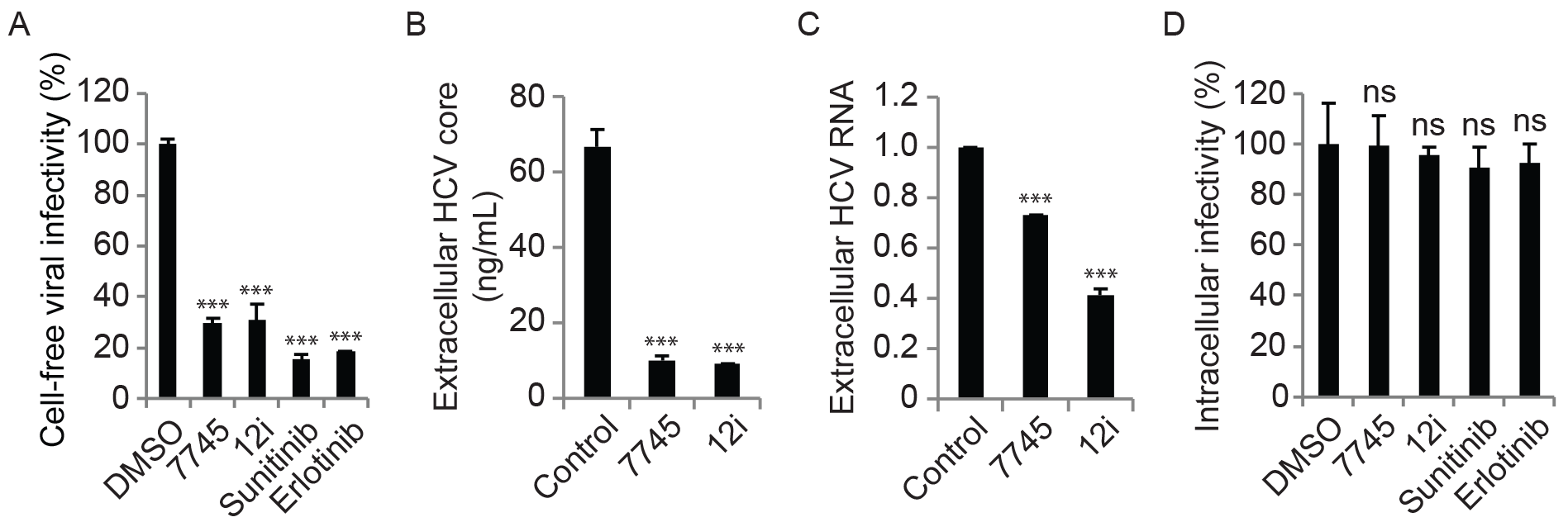

Supplement: FIG S3 [file mbo002183778sf3.tif]
