# Supplementary material for: Interactions between the Hepatitis C Virus Nonstructural 2 Protein and Host Adaptor Proteins 1 and 4 Orchestrate Virus Release
Source: mBio. 2018 Mar 13;9(2):e02233-17. doi: 10.1128/mBio.02233-17 (PMC5850324; doi:10.1128/mBio.02233-17)

Text S1

Supplemental Materials and Methods

Plasmids. ORFs encoding AP-1B, AP-2, AP-3, AP-4, and AP-5 μ subunits were selected from the Human ORFeome library of cDNA clones (Open biosystems) (1) and recombined into either pCherry or pGLuc vectors using Gateway technology (Invitrogen). ORF encoding the AP-1A subunit was obtained from Addgene and cloned into the same expression vectors described above. ORFs encoding HCV proteins were amplified from described vectors (2) and recombined into pGLuc vector(3). pFL-J6/JFH(p7-Rluc2A) (2), J6/H77NS2/JFH(NS2-IRES-nsGluc2AUbi) (4) and J6/H77NS2/JFH (5) were a gift from Dr. Rice. pH77S.3/GLuc2A was a gift from Dr. Lemon (6). GFP-LC3 (7), HCV TC-core (8, 9), and T144A AP-1 plasmids (9) were previously described. ShRNA-resistant WT AP-1B and AP-4 were cloned by introducing a wobble mutation within the site targeted by the respective shRNA. Mutations were introduced by site-directed mutagenesis using the QuikChange kit (Stratagene). pLentiCRISPR V2 plasmid was from Addgene (plasmid# 52961). Primer sequences will be provided upon request.

Reagents. The following reagents were used: sunitinib malate (Selleckchem), erlotinib (LC Laboratories), PIK93 (Tocris), siImporter (Millipore), Lipofectamine2000 (Invitrogen). 12i was synthesized by the Herdewijn laboratory (9, 10). 7745 was synthesized by ACME Bioscience, Inc (9). Mouse anti RAB11 antibody was from BD Transduction Lab.

Antibodies. Rabbit anti-AP4M1 (GeneTex), anti-AP1M1, AP1M2 or AP2M1 (Abcam), and anti-GLuc (New England BioLabs) antibodies, or mouse anti-NS5B (BioFront Technologies), and anti-beta actin (Sigma-aldrich) antibodies. Rabbit monoclonal anti-NS2 antibodies were previously described (3).

RNA interference (RNAi). Huh7.5 cells were transduced with lentiviral particles harboring shRNAs (Sigma). AP1M1, AP1M2, AP4M1 and AP2M1 were silenced via transduction with shRNA-expressing lentivirus (catalogue numbers: TRCN0000218336, TRCN0000141187, TRCN0000059718, TRCN0000060239 or Mission pLKO.1-puro non-mammalian shRNA control; Sigma-aldrich) and selection on 1 μg/ml puromycin. AP1M1 shRNA targeted a non-translated region whereas the other shRNAs targeted coding sequences.

Protein fragment complementation assays (PCAs). PCAs in mammalian cells were conducted as described (3, 11, 12). Briefly, combinations of plasmids encoding prey (A) and bait (B) proteins, each fused to a fragment of the *Gaussia* luciferase protein (GLuc1 and GLuc2) or control vectors were cotransfected into 293T cells plated in 96-well plates in triplicates. At 24 hours post-transfection, cells were lysed and subjected to luciferase assays (Promega). Results were expressed as normalized luminescence ratios (NLR): the average signal in cells transfected with GLuc1-A and GLuc2-B divided by the average signal in wells transfected with GLuc1-A and an empty GLuc2 vector and those transfected with GLuc2-B and an empty GLuc1 vector.

Live cell imaging. Huh7.5 cells were infected with concentrated HCV TC-core at MOI of 1 for 24 hours, then transfected with either AP-1A-, AP-1B-, AP-2-, or AP-4-mCherry using Lipofectamine2000 (Invitrogen) and seeded onto collagen coated 35 mm fluorodishes (World Precision Instruments)(8, 9). At 72 hours post-infection, cells were labeled with biarsenical dye (1.25 μM) in Opti-MEM at 37ºC for 30 minutes, then washed three times with 1X BAL (2,3-dimercapto-1-propanol) wash buffer (Invitrogen) supplemented with 500 μM EDT in Opti-MEM. The cells were washed and incubated in prewarmed imaging media (DMEM-F12, Invitrogen) supplemented with 10% fetal bovine serum, 0.1 mM nonessential amino acids, 1% penicillin-streptomycin, and 25 mM HEPES. When specified, TC-core infected cells were incubated with DMSO or PIK93 (0.5 μM) for 3 hours beginning at 72 hours post-infection. Timelapse images were taken using a Leica SP5 II AOBS Tandem Scanner Spectral confocal

microscope with a 100X 1.46 oil objective and a heated (37^oC) chamber. An average of 5 movies representing individual cells with trackable 15-30 puncta each were recorded per sample with sequential frames taken every 2 seconds. Individual core puncta run lengths and transport velocities were calculated using the Manual Tracking plugin for Image J, measuring the distance traveled (in any direction) between frames for a respective TC-core puncta.

Generation of AAK1- and GAK–knockout cell lines using CRISPR/Cas9. Single guide RNAs (sgRNA): AAK1: 5' CACCGCGGGCGACAGCAGGTCACAG and 5′ AAACCTGTGACCTGCTGTCGCCCGC site. GAK; 5' CACCGGCGACCAGAGTGACTTCGTG and 5' AAACCACGAAGTCACTCTGGTCGCC. The annealed oligos were cloned into pLentiCRISPR V2 plasmid following its digestion with FastDigest Esp3I (Thermo Fisher Scientific), as described (13). These plasmids and a pLentiCRISPR V2 non-targeting (NT) control plasmid were cotransfected individually along with the packaging plasmids pCMV-R8.91 pMD.G (Addgene) into HEK293-F T cells (ATCC) for preparation of the lentiviruses. CRISPR sgRNA lentivirus targeting AAK1 or GAK and CRISPR NT control lentivirus were transduced individually into Huh7.5 cells followed by selection in 2 μg/mL puromycin (InvivoGen). Single cell-derived clones were used for the binding experiments.

Co-immunoprecipitations. Co-IPs in membrane fractions derived from HCV RNA or GLuc-NS2 and AP-4 transfected cells were carried out as previously described (3, 12). ~20×10^6 Huh7.5 cells transfected with HCV J6/JFH RNA or GLuc-NS2 and AP-4 were collected by trypsinization 3 days post-transfection, washed once with PBS and incubated with 1mM dithiobis-succinimidyl-propionate (DSP) crosslinker (Pierce) solution for 2 hours on ice to allow covalent binding of the already bound interacting proteins. Tris (pH 7.5) was added at 20 mM for 15 minutes to quench unreacted DSP. Cells were washed once with PBS, resuspended in HME buffer (20 mM HEPES (pH 7.4), 1 mM EDTA, 2 mM MgCl2) supplemented with phenylmethylsulfonyl fluoride to a final concentration of 1 mM and a protease inhibitors cocktail (Sigma). Cells were lysed by two freeze-thaw cycles in dry ice/ethanol and then passaged through a 27.5-gauge needle 10 times. Nuclei were removed by centrifugation at 250×g for 10 minutes, and the postnuclear supernatant was subjected to ultracentrifugation at 100,000×g for 30 minutes. All steps were done at 4°C. Membrane pellets were resuspended in 100 µl HME buffer. TDB buffer (2.5% Triton X-100, 25 mM triethanolamine-Cl (pH 8.6), 20 mM NaCl, 5 mM EDTA, 0.2% NaN3) was added to a final volume of 1 ml. Samples were incubated overnight with protein G magnetic beads (Dynabeads, Life Technologies) crosslinked with either anti-AP, -NS2, -NS5B antibodies or IgG controls via bis(sulfosuccinimidyl)suberate (BS^3) crosslinker (Sulfo-DSS, Thermo Scientific Pierce). Following extensive washes, bound proteins were eluted from the beads by adding SDS sample buffer and heating for 10 minutes. Immunoprecipitates were analyzed by Western blotting.

Supplemental References

1. **Rual JF, Hirozane Kishikawa T, Hao T, Bertin N, Li S, Dricot A, Li N, Rosenberg J, Lamesch P, Vidalain PO, Clingingsmith T, Hartley J, Esposito D, Cheo D, Moore T, Simmons B, Sequerra R, Bosak S, Doucette-Stamm L, Le Peuch C, Vandenhaute J, Cusick M, Albala J, Hill D, Vidal M.** 2004. Human ORFeome version 1.1: a platform for reverse proteomics. Genome research **14:**2128-2135.
2. **Murray CL, Jones CT, Tassello J, Rice CM.** 2007. Alanine Scanning of the Hepatitis C Virus Core Protein Reveals Numerous Residues Essential for Production of Infectious Virus. J Virol **81:**10220-10231.
3. **Barouch-Bentov R NG, Xiao F, Beer M, Bekerman E, Schor S, Campbell J, Boonyaratanakornkit J, Lindenbach B, Lu A, Jacob Y, Einav S. .** 2016. Hepatitis C virus proteins interact with the endosomal sorting complex required for transport

machinery via ubiquitination to facilitate viral envelopment. mBio **7** e01456-01416. doi:01410.01128/mBio.01456-01416.

4. **Jones CT, Murray CL, Eastman DK, Tassello J, Rice CM.** 2007. Hepatitis C Virus p7 and NS2 Proteins Are Essential for Production of Infectious Virus. J Virol **81:**8374-8383.
5. **Dentzer TG, Lorenz IC, Evans MJ, Rice CM.** 2009. Determinants of the Hepatitis C Virus Nonstructural Protein 2 Protease Domain Required for Production of Infectious Virus. Journal of Virology **83:**12702-12713.
6. **Shimakami T, Welsch C, Yamane D, McGivern D, Yi M, Zeuzem S, Lemon SM.** 2011. Protease Inhibitor-Resistant Hepatitis C Virus Mutants with Reduced Fitness from Impaired Production of Infectious Virus. Gastroenterology **140:**667-675.
7. **Heaton NS, Randall G.** 2010. Dengue Virus-Induced Autophagy Regulates Lipid Metabolism. Cell Host & Microbe **8:**422-432.
8. **Coller K, Heaton N, Berger K, Cooper J, Saunders J, Randall G.** 2012. Molecular determinants and dynamics of hepatitis C virus secretion. PLoS Pathog **8:**e1002466-e1002466.
9. **Bekerman E, Neveu G, Shulla A, Brannan J, Pu S-Y, Wang S, Xiao F, Barouch-Bentov R, Bakken RR, Mateo R, Govero J, Nagamine CM, Diamond MS, De Jonghe S, Herdewijn P, Dye JM, Randall G, Einav S.** 2017. Anticancer kinase inhibitors impair intracellular viral trafficking and exert broad-spectrum antiviral effects. The Journal of Clinical Investigation **127**.
10. **Kovackova S, Chang L, Bekerman E, Neveu G, Barouch-Bentov R, Chaikuad A, Heroven C, Šála M, De Jonghe S, Knapp S, Einav S, Herdewijn P.** 2015. Selective Inhibitors of Cyclin G Associated Kinase (GAK) as Anti-Hepatitis C Agents. Journal of Medicinal Chemistry **58:**3393-3410.
11. **Cassonnet P, Rolloy, C., Neveu, G., Vidalain, P.O., Chnatier, T., Pellet, J., Jones,L., Muller, M., Demeret, C., Gaud, G., Vuillier, F., Lotteau, V., Tangy, F., Favre, M., Jacob, Y.** 2011. Benchmarking a luciferase complementation assay for detecting protein complexes. Nat Methods **8:**990-992.
12. **Neveu G, Barouch-Bentov R, Ziv-Av A, Gerber D, Jacob Y, Einav S.** 2012. Identification and Targeting of an Interaction between a Tyrosine Motif within Hepatitis C Virus Core Protein and AP2M1 Essential for Viral Assembly. PLoS Pathog **8:**e1002845.
13. **Sanjana NE, Shalem O, Zhang F.** 2014. Improved vectors and genome-wide libraries for CRISPR screening. Nat Meth **11:**783-784.

Figure 1C

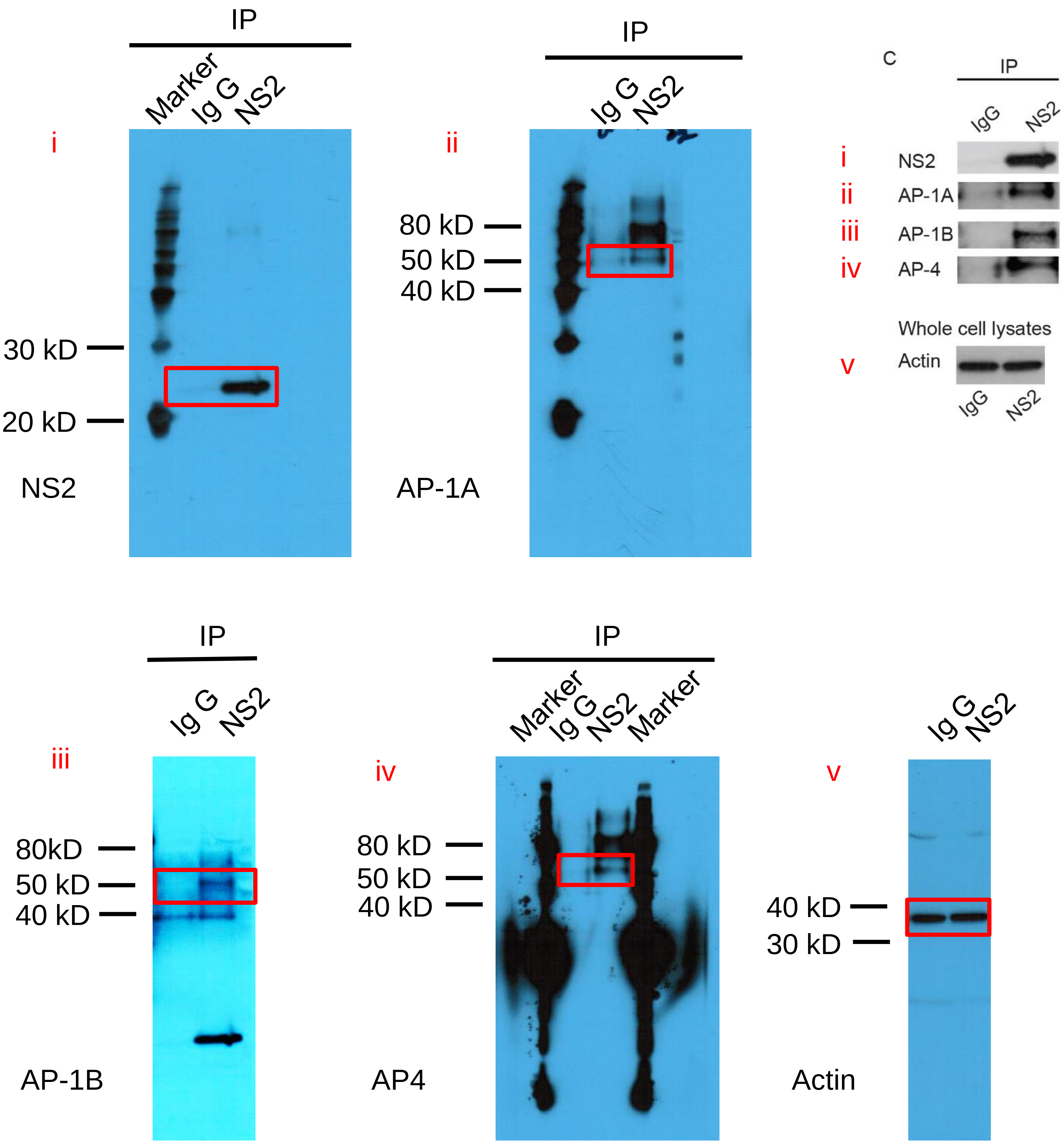
IP
Marker
Ig G
NS2
i
30 kD
20 kD
NS2
ii
80 kD
50 kD
40 kD
AP-1A
C
IP
IgG
NS2
i
NS2
ii
AP-1A
iii
AP-1B
iv
AP-4
Whole cell lysates
v
Actin
iii
80kD
50 kD
40 kD
AP-1B
iv
Marker
80 kD
50 kD
40 kD
AP4
v
40 kD
30 kD
Actin

Figure 1D

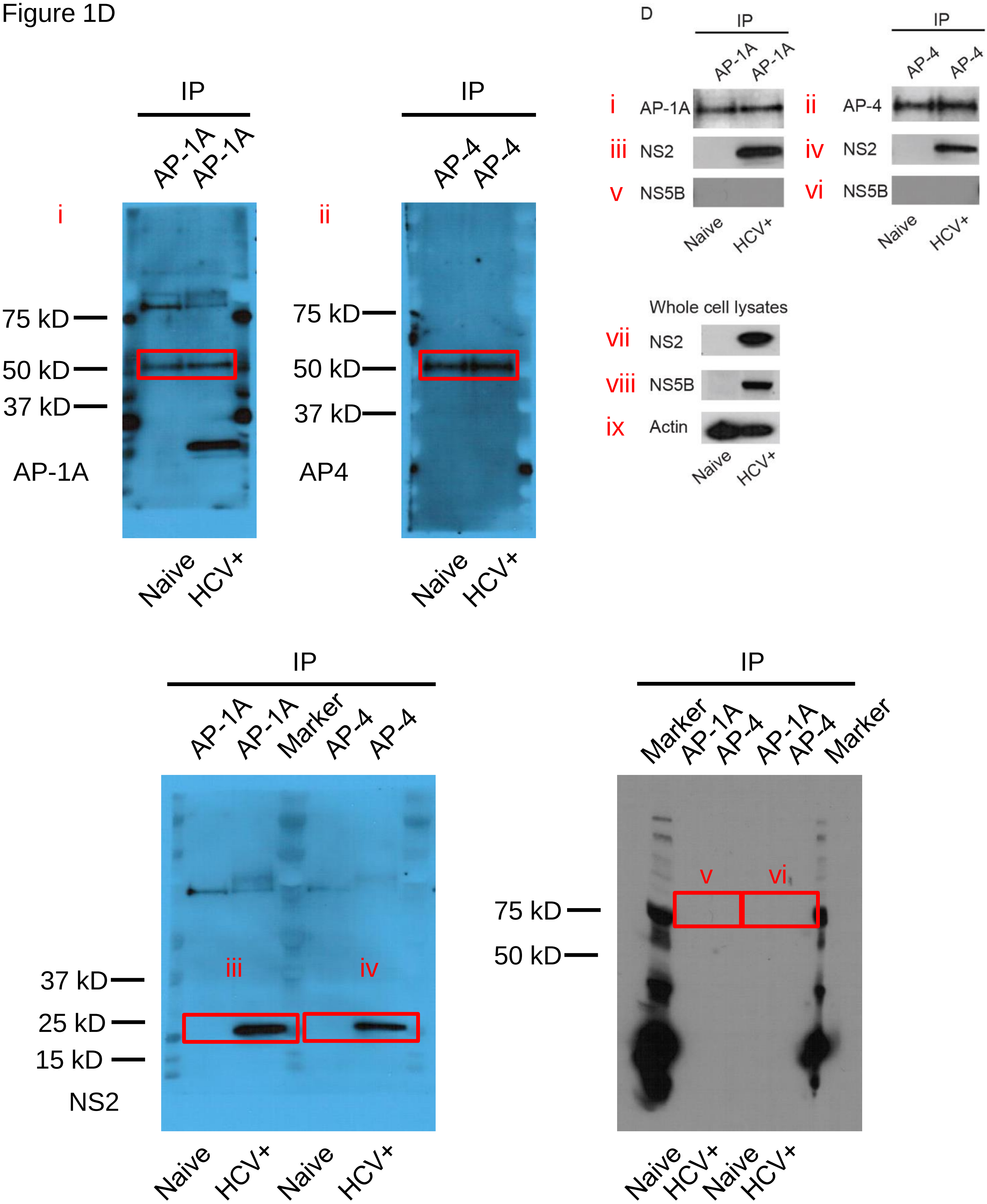

D
IP
AP-1A
AP-1A
IP
AP-4
AP-4
i
AP-1A
ii
AP-4
iii
NS2
iv
NS2
v
NS5B
vi
NS5B
Naive
HCV+
Naive
HCV+
Whole cell lysates
vii
NS2
viii
NS5B
ix
Actin
Naive
HCV+
IP
AP-1A
AP-1A
IP
AP-4
AP-4
i
ii
75 kD
50 kD
37 kD
75 kD
50 kD
37 kD
AP-1A
AP4
Naive
HCV+
Naive
HCV+
IP
AP-1A
AP-1A
Marker
AP-4
AP-4
iii
iv
37 kD
25 kD
15 kD
NS2
Naive
HCV+
Naive
HCV+
IP
Marker
AP-1A
AP-4
AP-1A
AP-4
Marker
v
vi
75 kD
50 kD
Naive
HCV+
Naive
HCV+

Figure 1D (continued)

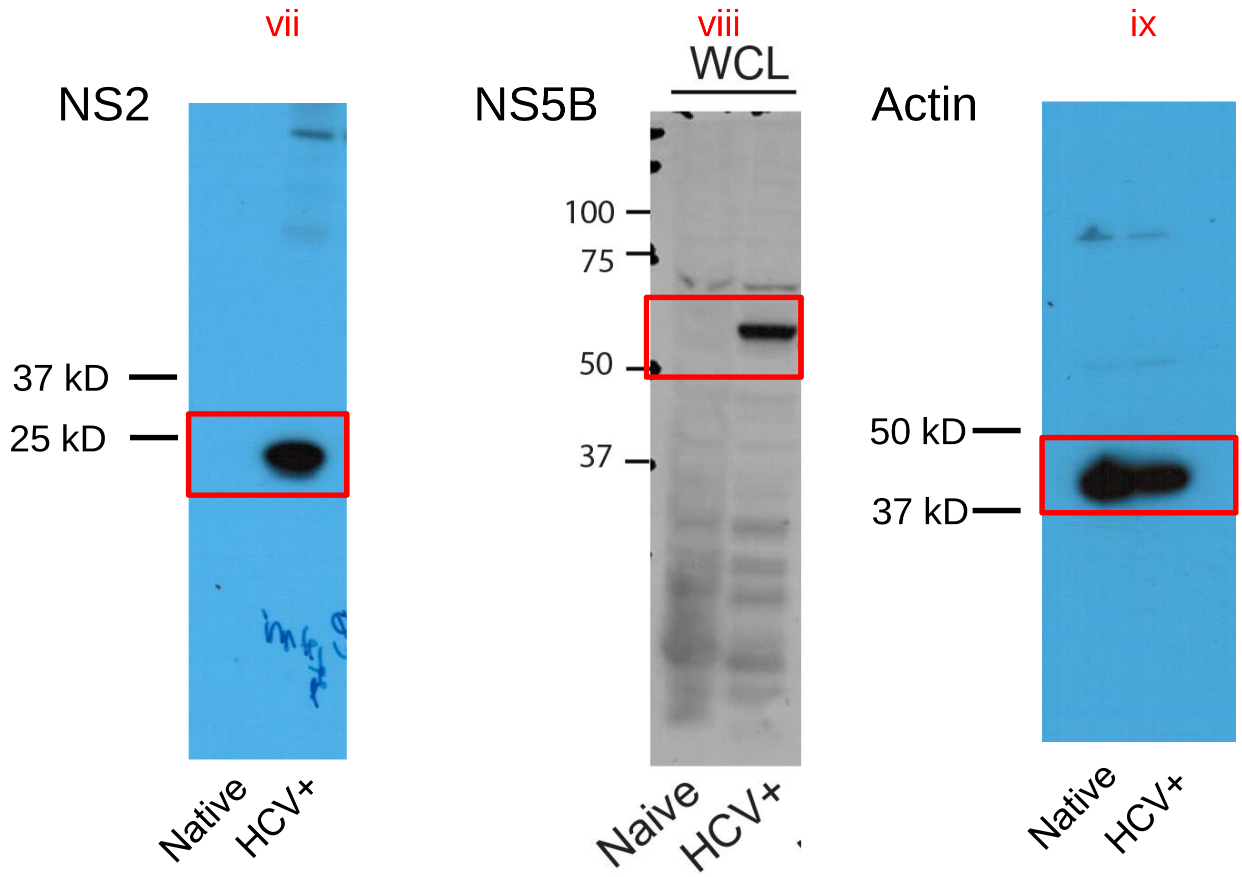

Figure 2B

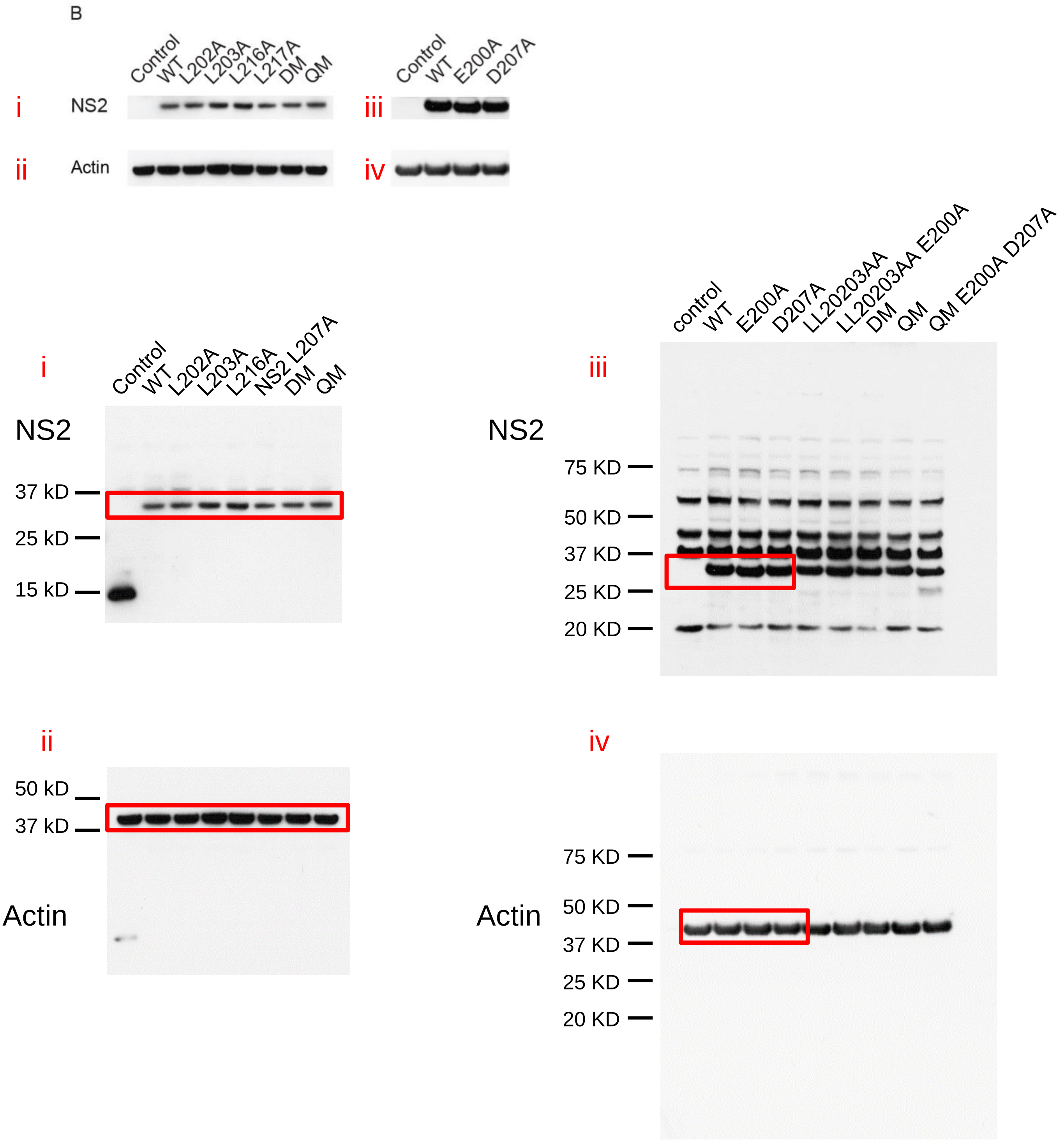
B
Control
WT
L202A
L203A
L216A
L217A
DM
QM
E200A
D207A
i
ii
iii
iv
NS2
Actin
NS2 L207A
37 kD
25 kD
15 kD
50 kD
control
LL20203AA
LL20203AA E200A
QM E200A D207A
75 KD
50 KD
37 KD
25 KD
20 KD

Figure 3A

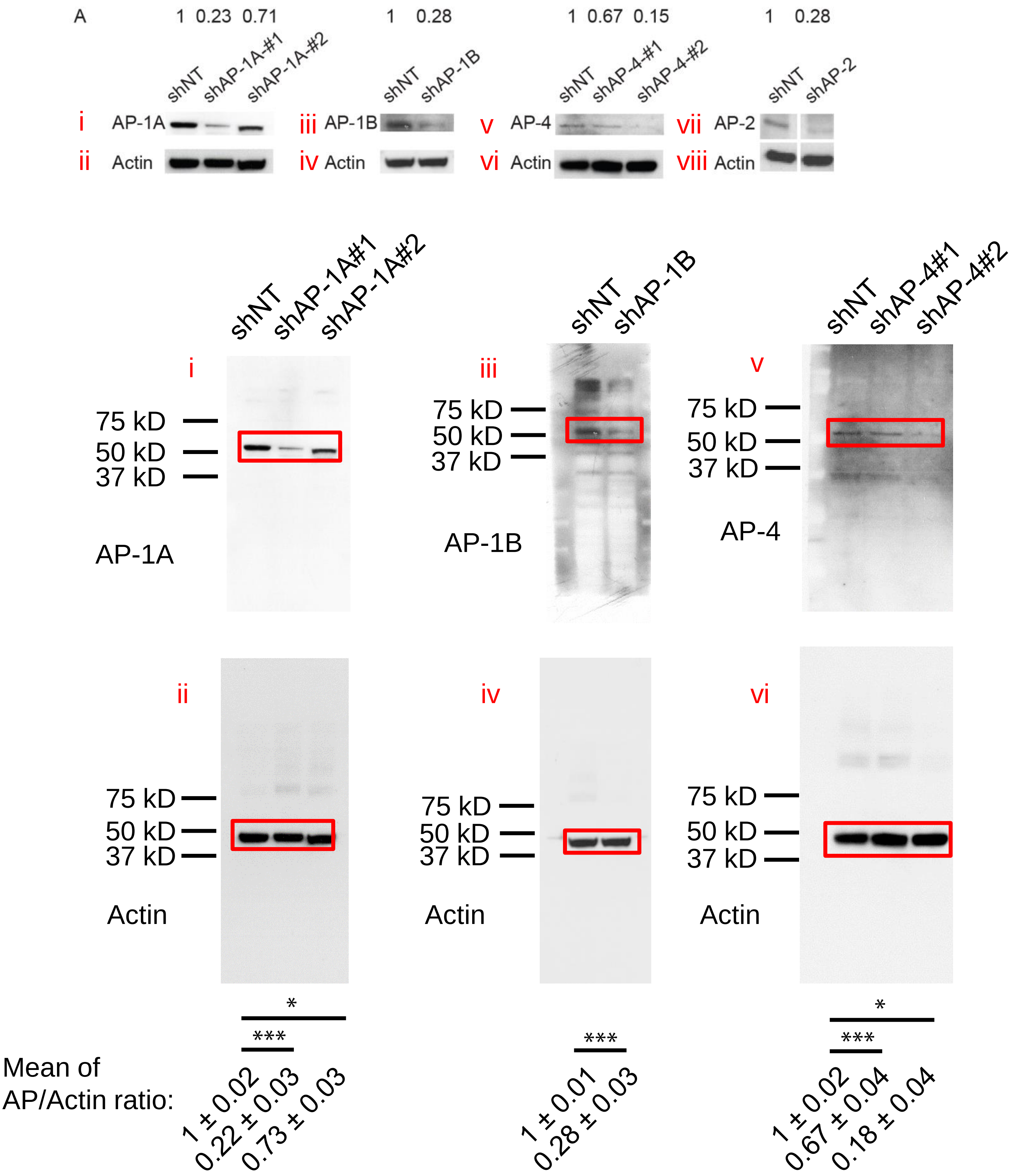
A
1 0.23 0.71
1 0.28
1 0.67 0.15
1 0.28
shNT shAP-1A-#1 shAP-1A-#2
shNT shAP-1B
shNT shAP-4-#1 shAP-4-#2
shNT shAP-2
i AP-1A
ii Actin
iii AP-1B
iv Actin
v AP-4
vi Actin
vii AP-2
viii Actin
shNT shAP-1A#1 shAP-1A#2
shNT shAP-1B
shNT shAP-4#1 shAP-4#2
i
iii
v
75 kD
50 kD
37 kD
AP-1A
AP-1B
AP-4
ii
iv
vi
Actin
*
***
Mean of AP/Actin ratio:
1 ± 0.02
0.22 ± 0.03
0.73 ± 0.03
1 ± 0.01
0.28 ± 0.03
1 ± 0.02
0.67 ± 0.04
0.18 ± 0.04

Figure 3A (continued)

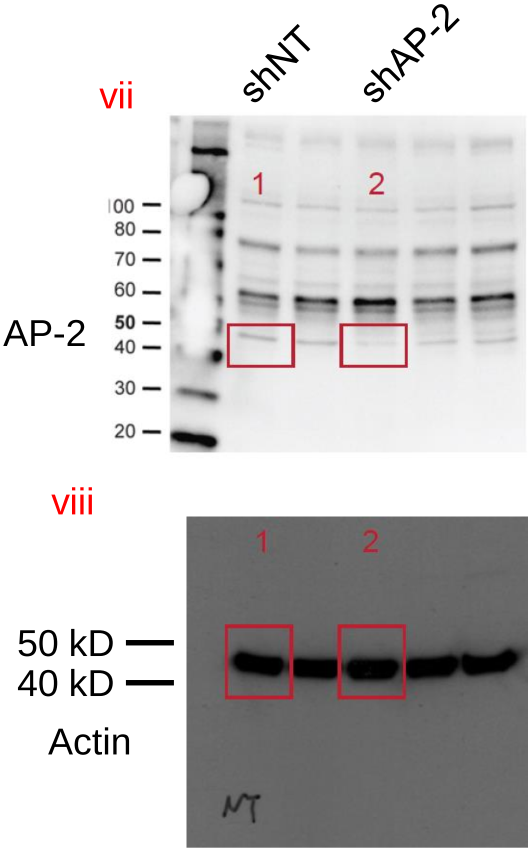

Mean of AP/Actin ratio:

1 ± 0.01

0.28 ± 0.03

***

Figure 3E

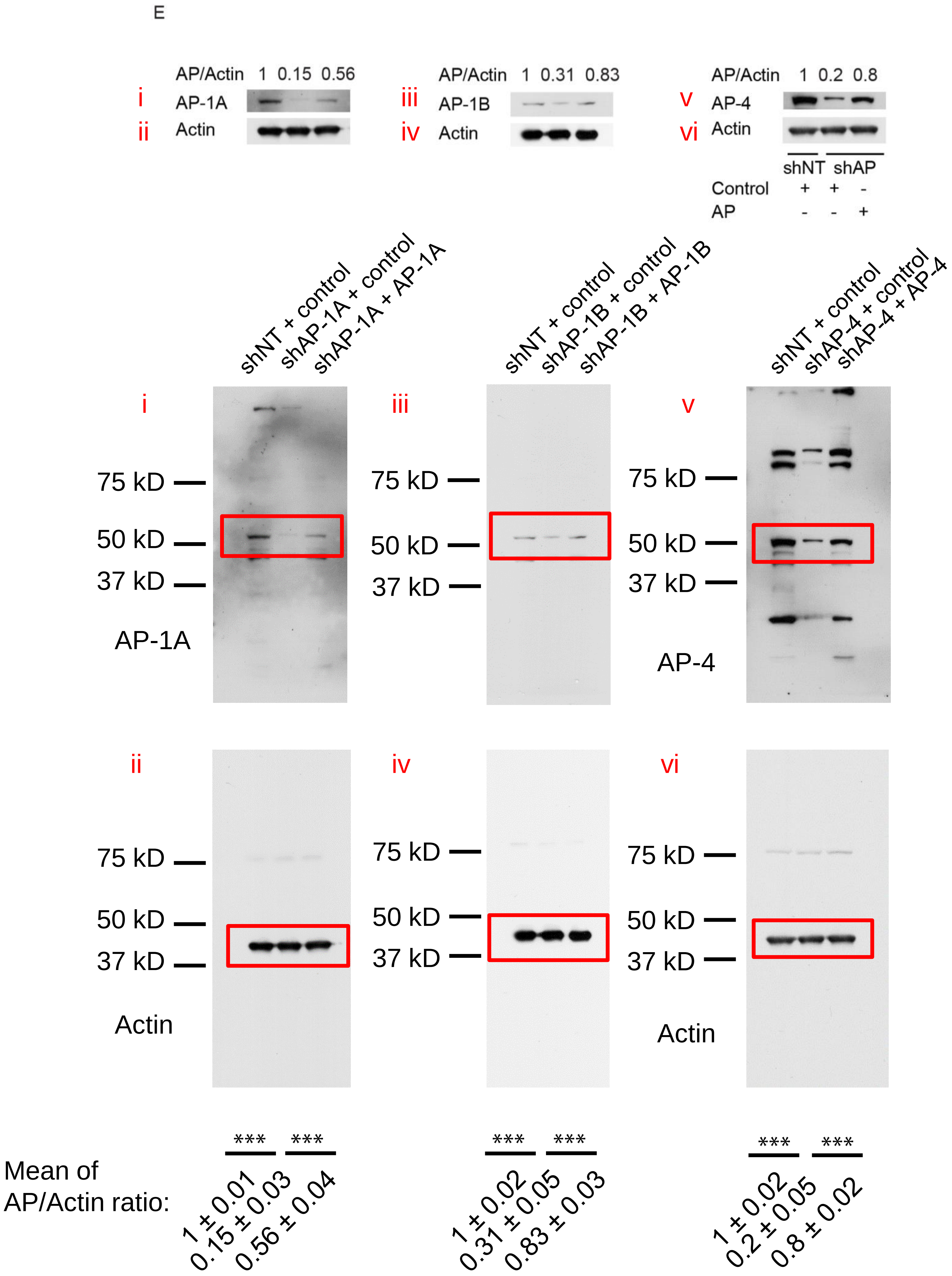

E
AP/Actin 1 0.15 0.56
i AP-1A
ii Actin
AP/Actin 1 0.31 0.83
iii AP-1B
iv Actin
AP/Actin 1 0.2 0.8
v AP-4
vi Actin
shNT shAP
Control + + -
AP - - +
shNT + control
shAP-1A + control
shAP-1A + AP-1A
shNT + control
shAP-1B + control
shAP-1B + AP-1B
shNT + control
shAP-4 + control
shAP-4 + AP-4
i
iii
v
75 kD
50 kD
37 kD
AP-1A
AP-4
ii
iv
vi
Actin
***
Mean of AP/Actin ratio:
1 ± 0.01
0.15 ± 0.03
0.56 ± 0.04
1 ± 0.02
0.31 ± 0.05
0.83 ± 0.03
1 ± 0.02
0.2 ± 0.05
0.8 ± 0.02

Figure 4F

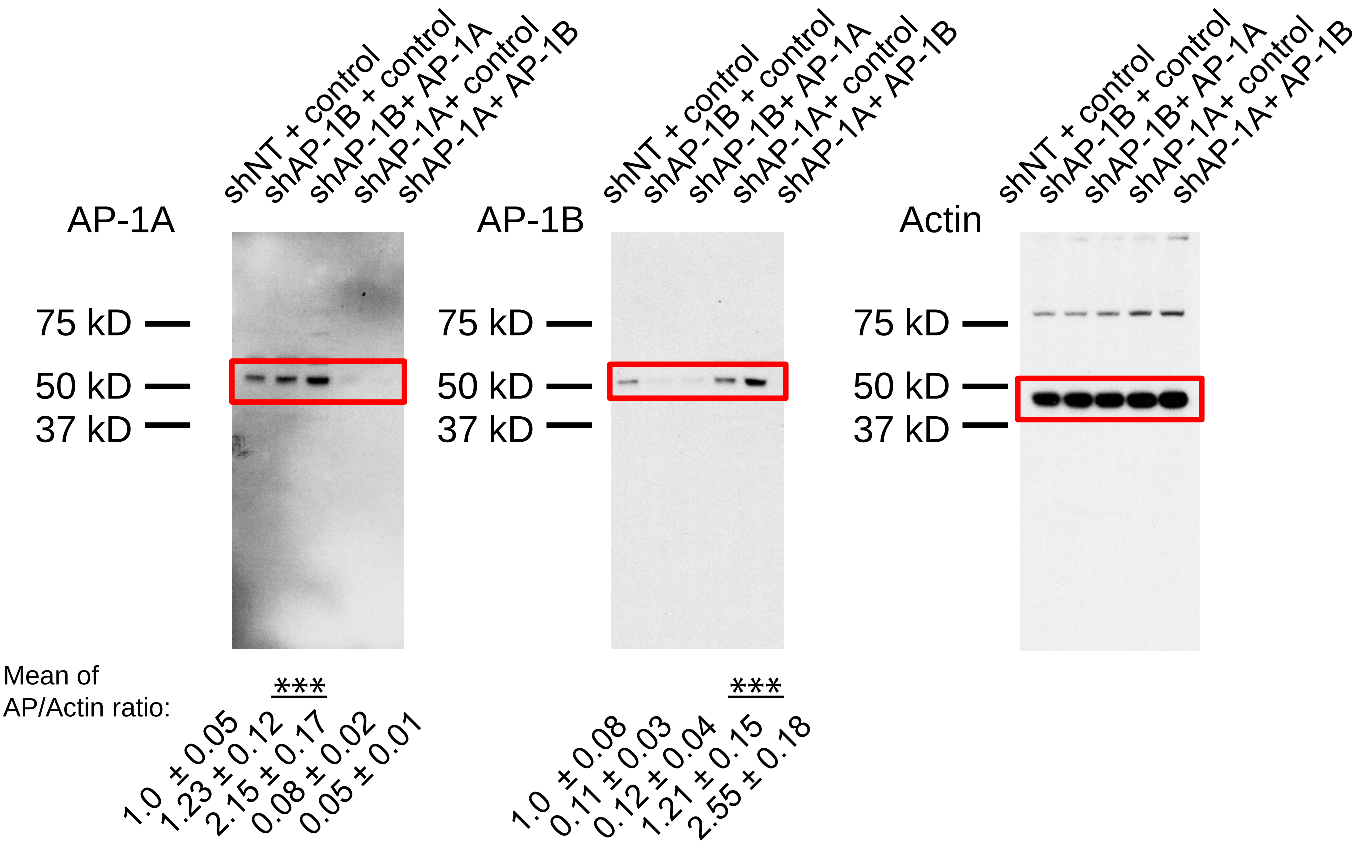

AP-1A
AP-1B
Actin
shNT + control
shAP-1B + control
shAP-1B+ AP-1A
shAP-1A+ control
shAP-1A+ AP-1B
75 kD
50 kD
37 kD
Mean of AP/Actin ratio:
***
1.0 ± 0.05
1.23 ± 0.12
2.15 ± 0.17
0.08 ± 0.02
0.05 ± 0.01
***
1.0 ± 0.08
0.11 ± 0.03
0.12 ± 0.04
1.21 ± 0.15
2.55 ± 0.18

Figure 6A

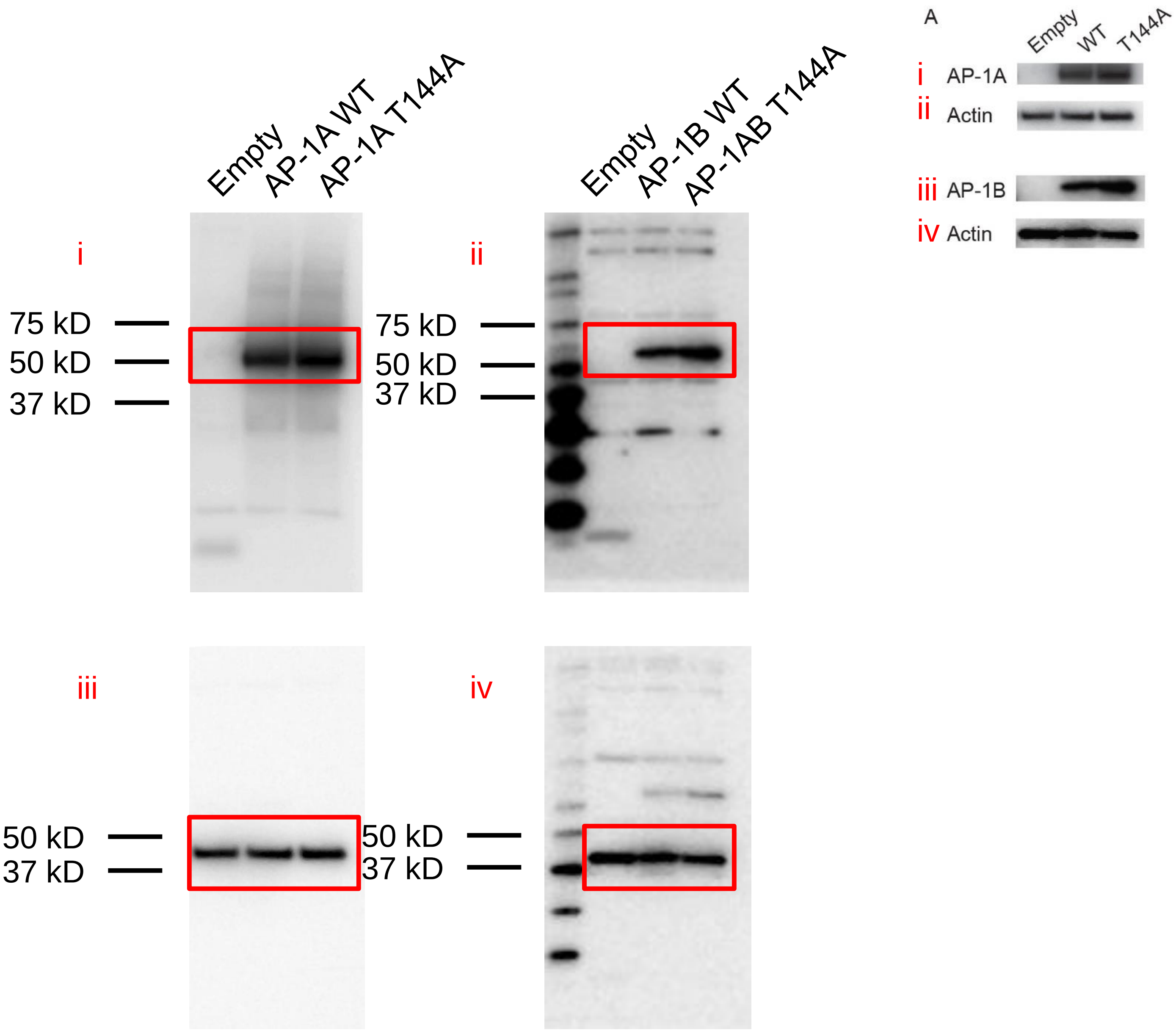

A
Empty
WT
T144A
i AP-1A
ii Actin
iii AP-1B
iv Actin
Empty
AP-1A WT
AP-1A T144A
Empty
AP-1B WT
AP-1AB T144A
i
ii
iii
iv
75 kD
50 kD
37 kD
75 kD
50 kD
37 kD
50 kD
37 kD
50 kD
37 kD

Figure 6D

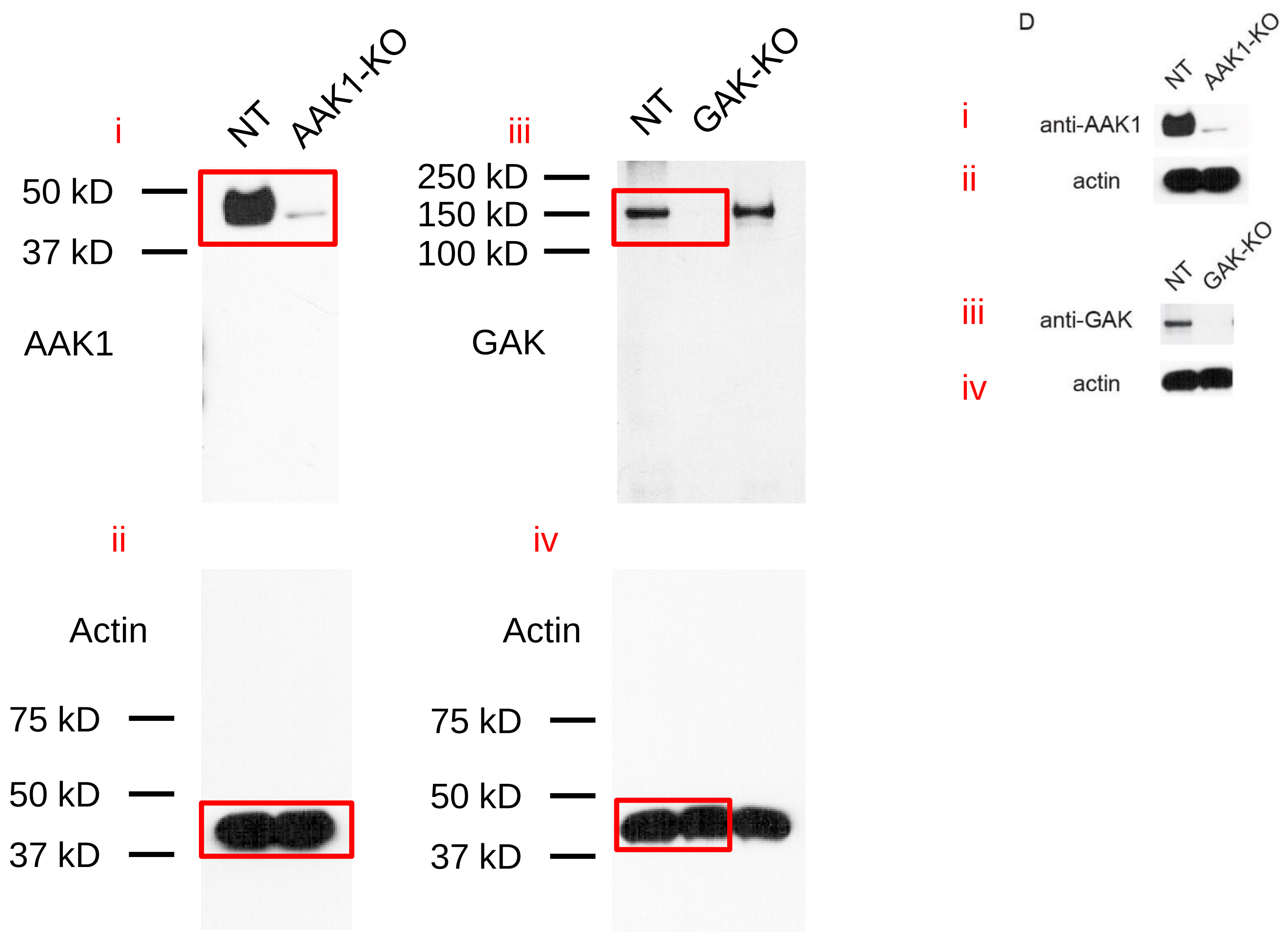
i
NT
AAK1-KO
50 kD
37 kD
AAK1
iii
NT
GAK-KO
250 kD
150 kD
100 kD
GAK
ii
Actin
75 kD
50 kD
37 kD
iv
Actin
75 kD
50 kD
37 kD
D
NT
AAK1-KO
i
anti-AAK1
ii
actin
NT
GAK-KO
iii
anti-GAK
iv
actin

Figure S1

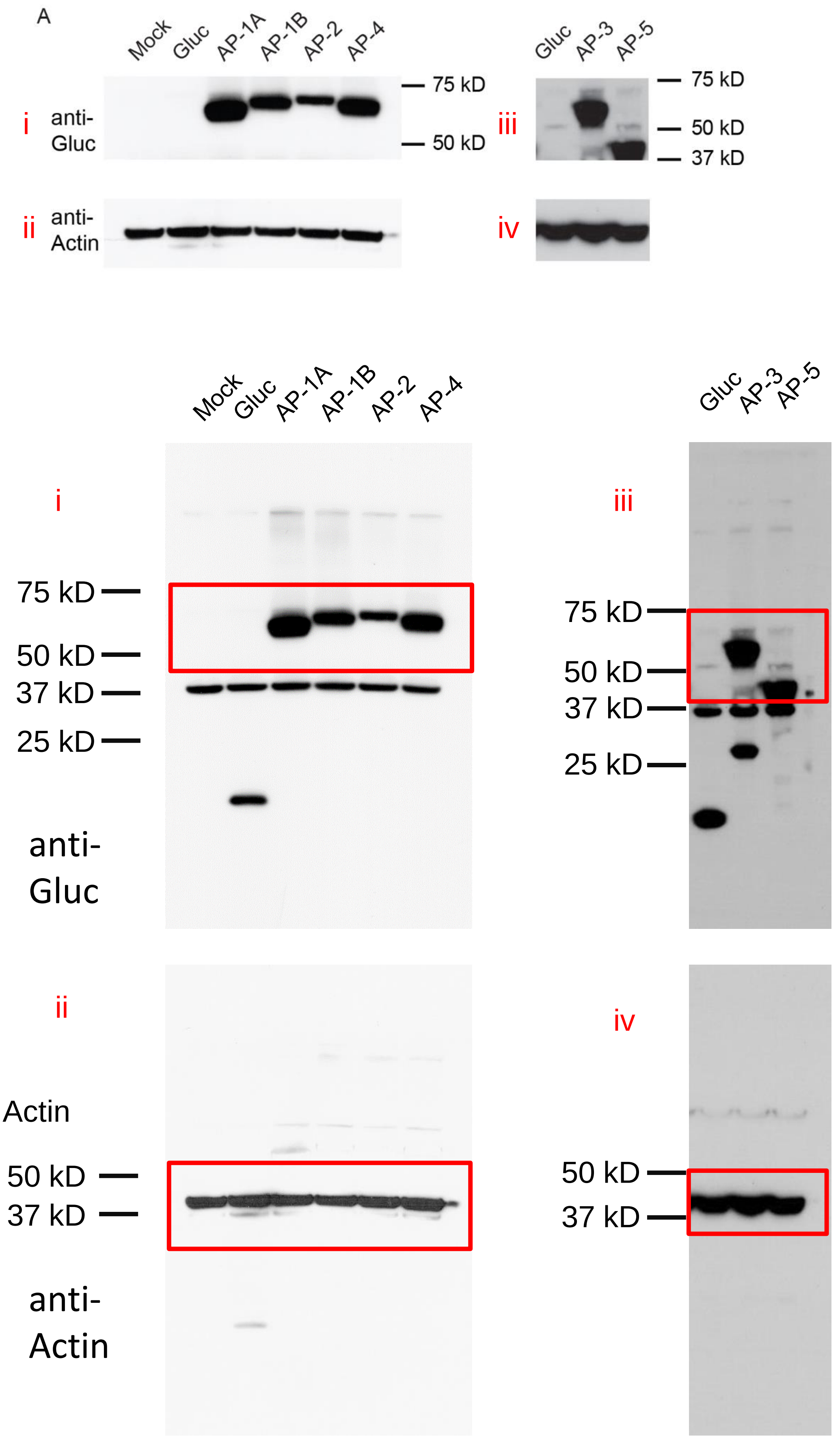
A
Mock
Gluc
AP-1A
AP-1B
AP-2
AP-4
i
anti-Gluc
75 kD
50 kD
ii
anti-Actin
Gluc
AP-3
AP-5
iii
75 kD
50 kD
37 kD
iv
Mock
Gluc
AP-1A
AP-1B
AP-2
AP-4
i
75 kD
50 kD
37 kD
25 kD
anti-Gluc
Gluc
AP-3
AP-5
iii
75 kD
50 kD
37 kD
25 kD
ii
Actin
50 kD
37 kD
anti-Actin
iv
50 kD
37 kD

Figure S1 continued

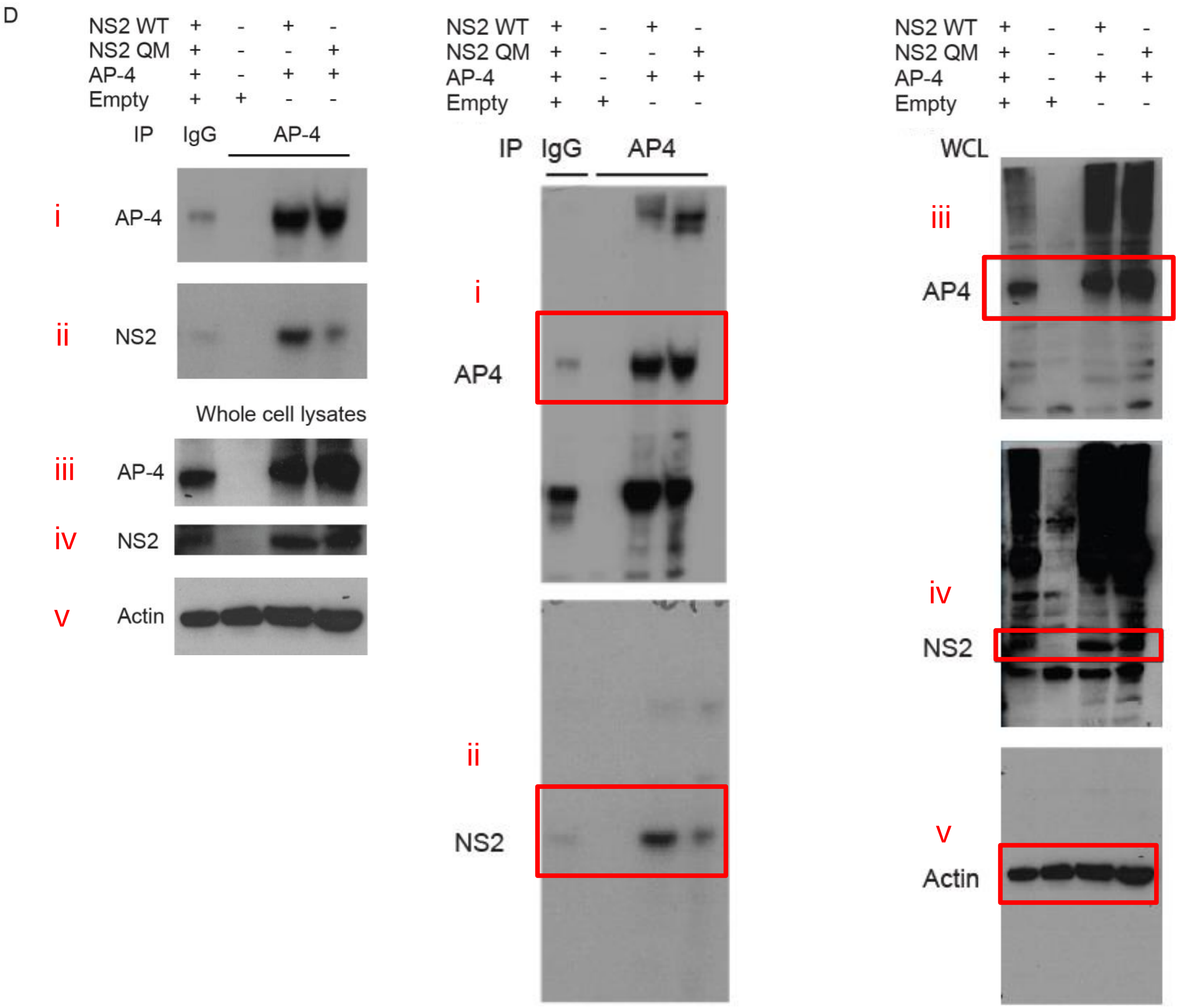

Supplement: TEXT S1 [file mbo002183778s1.pdf]
